# Supplementary material for: Children’s family income is associated with cognitive function and volume of anterior not posterior hippocampus
Source: Nat Commun. 2020 Aug 12;11:4040. doi: 10.1038/s41467-020-17854-6 (PMC7423938; doi:10.1038/s41467-020-17854-6)
Supplement: Supplementary file 1 — Supplementary Information [file 41467_2020_17854_MOESM1_ESM.pdf]

Supplementary Information

Children's family income is associated with cognitive function and volume of anterior not posterior hippocampus

Decker et al.

Supplementary Methods

Correcting for intracranial volumes. Regional hippocampal volumes were corrected for individual differences in intracranial volume (ICV). The adjustment for ICV was performed using a regression-based approach. The formula for adjusting volumes is as follows:

(1) $\text{Volume}_{\text{adjusted}} = \text{volume}_{\text{raw}} - (b^{*}(\text{ICV}_i - \text{mean ICV}))$

In this formula, $\text{Volume}_{\text{adjusted}}$ is the volume of hippocampal subregions after adjusting for an individual's ICV. $\text{Volume}_{\text{raw}}$ is the raw hippocampal subregion volume, *b* is the slope when ICV is regressed onto $\text{volume}_{\text{raw}}$, ICV_i is the ICV for individual i and mean ICV is the mean ICV for the entire sample. All analyses were performed using hippocampal subregion volumes that had been adjusted for ICV.

Testing whether the relationship between intracranial volumes differed by age and sex. Prior to adjusting hippocampal volumes for intracranial volume (ICV) we tested whether the relationship between hippocampal subregions and ICV differed across ages and between sexes. We separated our sample into 4 age quartiles: younger children (3-7 years old), older children (8-12 years old), adolescents (13-17 years old), and young adults (18-21 years old). We then ran 4 separate linear regression models (e.g., subregion ~ ICV * age * sex) to test whether age or sex moderated the link between ICV and right and left anterior and posterior hippocampal volumes. We found that age, but not sex moderated ICV-hippocampal volume relationships. The pattern of age effects differed depending on the region, and therefore we report these relationships in separate sections below.

Right anterior hippocampal volumes. Right anterior hippocampal volume-ICV relationships did not differ between younger and older children ($b = -0.00006$, $SE = 0.0001$, $t(694) = -0.48$, $p = .628$, $r = 0.02$). Younger children differed from adolescents

(b = −0.0003, SE = 0.0001, t(694) = −2.52, p = .012, r = 0.10), but not adults (b = −0.0002, SE = 0.0001, t(694) = −1.56, p = .119, r = 0.06). Older children differed from adolescents (b = −0.0003, SE = 0.00009, t(694) = −2.71, p = .007, r = 0.10), but not adults (b = −0.0002, SE = 0.0001, t(694) = −1.40, p = .161, r = 0.05). Adolescents and young adults did not differ from each other (*b = 0.0001, SE = 0.0001, t(694) = 0.93, p = .353, r = 0.04)*. Since younger and older children did not differ from each other, and adolescents and adults did not differ from each other, we combined younger and older children into one group and adolescents and young adults into another and performed the ICV adjustments on these two groups separately.

Left anterior hippocampal volumes. The relationship between left anterior hippocampal volumes and ICV did not differ between young children and older children (b = 0.00003, SE = 0.0001, t(694) = 0.28, p = .777, r = 0.01), adolescents (b = −0.0002, SE = 0.0001, t(694) = −1.26, p = .208, r = 0.05) or young adults (b = −0.00003, SE = 0.0001, t(694) = −0.23, p = .819, r = 0.009). However, older children differed from adolescents (b = −0.0002, SE = 0.00009, t(694) = −2.04, p = .042, r = 0.08), but not young adults (b = −0.00006, SE = 0.0001, t(694) = −0.61, p = .544, r = 0.02). Adolescents and young adults did not differ from each other (*b = 0.0001, SE = 0.0001, t(694) = 1.14, p = .254, r = 0.04*). Therefore, we combined younger and older children into one group and adolescents and young adults into another and performed the ICV adjustments on these two groups separately.

Right posterior hippocampal volumes. The relationship between right posterior hippocampal volume and ICV did not differ between younger children and older children (b = −0.00008, SE = 0.0001, t(694) = −0.75, p = .451, r = 0.03), younger children and adolescents (b = −0.0001, SE = 0.0001, t(694) = −0.95, p = .342, r = 0.04), or younger children and adults (b = −0.00002, SE = 0.0001, t(694) = −0.16, p = .872, r = 0.006). Older children did not differ in their relationship from adolescents (b = −0.00002, SE = 0.00008, t(694) = −0.27, p = .784, r = 0.01) or young adults (b = 0.00006, SE = 0.00009, t(694) = 0.65, p = .513, r = 0.02). Adolescents and young adults did not differ in this

relationship from each other ($b = 0.00008$, $SE = 0.00009$, $t(694) = 0.88$, $p = .382$, $r = 0.03$). Therefore, we combined all age groups to perform the ICV adjustments.

Left posterior hippocampal volumes. The relationship between left posterior hippocampal volumes and ICV did not differ between young children and older children ($b = -0.0002$, $SE = 0.0001$, $t(694) = -1.84$, $p = .07$, $r = 0.07$), young children and adolescents ($b = -0.0001$, $SE = 0.0001$, $t(694) = -1.32$, $p = .187$, $r = 0.05$) and young children and young adults ($b = -0.0002$, $SE = 0.0001$, $t(694) = -1.59$, $p = .113$, $r = 0.06$). Older children did not differ in this relationship from adolescents ($b = 0.00005$, $SE = 0.00008$, $t(694) = 0.65$, $p = .517$, $r = 0.02$) or young adults ($b = 0.000009$, $SE = 0.00009$, $t(694) = 0.10$, $p = .924$, $r = 0.004$). Adolescents and young adults did not differ in this relationship from each other ($b = -0.00004$, $SE = 0.0001$, $t(694) = -0.46$, $p = .647$, $r = 0.02$). Therefore, we combined all age groups to perform the ICV adjustments.

Calculating how many years apart the lowest and highest income bracket differ in terms of cognitive scores. In order to test how far apart the lowest and higher income bracket were cognitively in units of age, we first converted our dependent variables (memory and vocabulary scores) into z scores, and ran 2 separate regression models, in which cognitive scores (in z score units) served as the dependent variable, and age, income, and sex served as predictors. From these models, we extracted the intercept for income, cognitive scores and age.

We next calculated the difference in cognitive scores between individuals in the lowest versus highest income bins (in units of z scores). To calculate cognitive scores for the lowest income bin, we multiplied the lowest income bin by the intercept for income, and then added this value to the intercept for cognition. A similar procedure was performed to calculate cognitive scores for individuals in the highest income bin. Once we had calculated cognitive scores for the lowest and highest income bins, we divided the difference by the intercept for age.

We note that because of the cross-sectional nature of the study, these results should be interpreted with caution. Future research would benefit from using

longitudinal data to measure the age-related trajectory of cognitive development and how memory differs in individuals from wealthy and low-income backgrounds.

Supplementary Note 1

Exploratory analyses testing whether age moderated volume-cognition relationships. We explored whether age moderated volume-cognition relationships by adding age as an interaction term to models assessing how anterior and posterior volumes relate to memory and vocabulary scores. There were no significant interactions between age and anterior hippocampal volumes to predict memory or vocabulary scores (ps > 0.05, Supplementary Tables 41 & 42). However, age moderated the relationship between posterior hippocampal volumes and memory scores ($b = -0.0007$, $SE = 0.0002$, $t(685) = -2.98$, $p = .003$, *p-adjusted* $= .04$, $r = 0.11$) and vocabulary scores ($b = -0.00007$, $SE = 0.00002$, $t(685) = -2.85$, $p = .005$, *p-adjusted* $= 0.04$, $r = 0.11$, Supplementary Tables 43 & 44).

To further investigate this interaction, we divided our sample into separate age bins (young children: 3-7, n=126; older children: 8-12, n=245; adolescents: 13-17, n=170; young adults: 18-21, n=149) and ran a regression model to test which age bins differed from each other. We found that adolescents had a more positive relationship between the posterior hippocampus and memory scores than did older children (interaction: b = 0.006, SE = 0.003, t(680) = 2.14, p = .032, r = 0.08). Examining the relationship between posterior volumes and memory scores in these groups separately revealed that the memory-volume relationships were not statistically significant in any group (young children: $b = -0.001$, $SE = 0.002$, $t(122) = -0.63$, $p = .530$, $r = 0.06$; older children: $b = -0.003$, $SE = 0.002$, $t(241) = -1.64$, $p = .102$, $r = 0.11$; adolescents: $b = 0.003$, $SE = 0.002$, $t(166) = 1.26$, $p = .209$, $r = 0.10$; young adults: $b = -0.0003$, $SE = 0.002$, $t(145) = -0.14$, $p = .887$, $r = 0.01$). Similarly, there were no relationships between the posterior hippocampus and vocabulary scores in any age group (young children: $b = -0.00009$, $SE = 0.0002$, $t(122) = -0.39$, $p = .698$, $r = 0.04$; older children: $b = 0.00002$, $SE = 0.0002$, $t(241) = 0.12$, $p = .902$, $r = 0.008$; adolescents, b = 0.0001, SE = 0.0002, t(166) = 0.46, p = .645, r = 0.04; young adults: $b = -0.0001$, $SE = 0.0003$, $t(145) = -0.52$, $p = .602$, $r = 0.04$). We note that our results might differ from previous studies

examining how age moderates volume-cognition relationships[70], however, owing to the large sample size in our study, we are well powered to detect significant effects.

Supplementary Note 2

Exploratory analyses testing whether nonlinear age transformations altered our findings. For models that included cognitive scores as the dependent variable, we controlled for the linear effect of age, but did not include nonlinear age terms as covariates. However, for completeness, we also tested whether nonlinear age transformations (quadratic, cubic) explained additional variance in cognitive scores. We found that the linear, quadratic and cubic age terms all explained significant variance in memory scores in the full sample (ps < .05). In contrast, the quadratic (but not cubic) age term explained additional significant variance in memory scores in the lower income sample ($p < .05$). Similarly, the quadratic (but not cubic) age term explained significant variance in vocabulary scores in the full sample, and in the lower income subsample (ps < .05). Therefore, to ensure the pattern of results were maintained, we re-fit the models in which cognitive scores were the dependent variable and included the relevant non-linear age transformations as covariates. We report the results from these models below.

Adding non-linear age terms to income-cognitive score models. Including nonlinear age transformations did not change the pattern of results for income-cognitive score models. Income significantly explained variance in memory scores in the full (b = 1.56, SE = 0.28, t(684) = 5.49, p < .001, r = 0.21) and lower income subsample (b = 2.06, SE = 0.45, t(394) = 4.58, p < .001, r = 0.22). Similarly, income significantly explained variance in vocabulary scores in the full (b = 0.27, SE = 0.03, t(685) = 8.90, p < .001, r = 0.32) and lower income subsample (b = 0.23, SE = 0.05, t(394) = 4.74, p < .001, r = 0.23).

Adding non-linear age terms to cognitive score-anterior hippocampal volume models. Including nonlinear age transformations did not change the pattern of results for models investigating relationships between anterior hippocampus and cognitive scores. The anterior hippocampus correlated with memory scores in the full sample (b = 0.002, SE =

0.0009, t(684) = 2.15, p = .032, r = 0.08) and lower income subsample (b = 0.003, SE = 0.001, t(394) = 2.39, p = .017, r = 0.12). The anterior hippocampus significantly correlated with vocabulary scores in the full sample (b = 0.0003, SE = 0.0001, t(685) = 3.23, p = .001, r = 0.12) and lower income subsample (b = 0.0003, SE = 0.0001, t(394) = 2.22, p = .027, r = 0.11).

Adding nonlinear age terms to mediation analysis. Similarly, after including nonlinear age transformations, anterior hippocampus marginally mediated income-gaps in memory scores in the full sample (p = 0.096) and the lower income subsample (p = 0.07). Anterior hippocampus significantly mediated income-relate gaps in vocabulary scores in the full sample (p = 0.007), and lower income subsample (p = 0.03).

Supplementary Tables

Supplementary Table 1. Family income data in the sample

Income bin	Recoded value	Number of participants	Cumulative sum
Less than $5000	4,500	22	22
$5,000 - $9,999	7,500	15	37
$10,000 - $19,999	15,000	46	83
$20,000-$29,999	25,000	38	121
$30,000 - $39,999	35,000	47	168
$40,000-$49,999	45,000	45	213
$50,000-$99,999	75,000	197	410
$100,000-149,999	125,000	155	565
$150,000-$199,999	175,000	72	637
$200,000-249,999	225,000	28	665
$250,000-$299,999	275,000	13	678
$300,000 and above	325,000	25	703

Note: Family income data were collected in bins and then recoded for analyses. The number of participants in each bin and the cumulative sum is detailed. After family income values were recoded, they were log transformed to approximate a normal distribution before being entered into statistical models.

Supplementary Table 2. Years of parental education in the sample

Parental education	Recoded value	Number of participants	Cumulative sum
Less than 7 years of school	6	4	4
7-9 years of school	8	6	10
10-11 years of school	10.5	16	26
High school graduate	12	113	139
Some college	14	132	271
4-year college graduate	16	303	574
Professional degree	18	118	692

Note: Parental education data were collected in bins and recoded for analysis. The number of participants in each bind and the cumulative sum is detailed below. Parental education reflects average parental education of both parents. In the case that education was only available for one parent, that one parents' years of education was reported.

Supplementary Table 3. Family income and episodic memory relationship

Variable	*b*	*t*	*p*	*Adjusted R^2*
				0.5096
Income	1.67	5.44	<0.001*** (<0.001***)	
Age	1.58	26.49	<0.001***	
Sex	0.05	0.17	0.869	

Note: A linear regression was used for this analysis (n=690 participants). Family income (in thousands of dollars) was log transformed, sex was effect-coded (female = -1, male = 1), and age was mean centered in the model. Episodic memory scores were missing from 13 of the 703 participants included in the volume analyses. FDR corrected *p* value is reported in brackets for the critical effect of interest.

Supplementary Table 4. Family income and vocabulary relationship

Variable	*b*	*t*	*p*	*Adjusted R^2*
				0.6648
Income	0.283	8.877	<0.001*** (<0.001***)	
Age	0.225	36.495	<0.001***	

Sex	0.106	3.499	<0.001***	

Note: A linear regression was used for this analysis (n=690 participants). Family income (in thousands of dollars) was log transformed, sex was effect-coded (female = -1, male = 1), and age was mean centered in the model. Vocabulary scores were missing from 13 participants of the 703 participants included in the volume analyses. FDR corrected p value is reported in brackets for the critical effect of interest.

Supplementary Table 5. Family income and anterior hippocampal volumes relationship

Variable	*b*	*t*	*p*	*Adjusted R^2*
				0.112
Income	43.2	3.55	<0.001*** (<0.001***)	
Age	11.31	4.38	<0.001***	
Sex	33.16	2.85	0.005**	
Scanner 1	−38.11	−1.32	0.188	
Scanner 2	−61.49	−2.08	0.038*	
Scanner 3	13.91	0.49	0.625	
Scanner 4	81.01	3.01	0.003**	
Scanner 5	19.86	0.55	0.582	
Scanner 6	−135.94	−4.85	<0.001***	

Note: A linear regression was used for this analysis (n= 703 participants). Age was mean centered, sex was effect-coded (female = -1, male = 1), scanner was dummy coded and family income (in thousands of dollars) was log transformed prior to being included in the model. FDR corrected *p* value is reported in brackets for the critical effect of interest.

Supplementary Table 6. Family income and posterior hippocampal volume relationship

Variable	*b*	*t*	*p*	*Adjusted R^2*
				0.119
Income	−14.88	-1.42	0.156 (0.16)	
Age	18.54	8.34	<0.001 ***	
Sex	−5.49	-0.55	0.58	
Scanner 1	−25.13	-1.01	0.31	
Scanner 2	49.06	1.6	0.11	
Scanner 3	18.24	0.79	0.43	
Scanner 4	3.45	0.03	0.98	
Scanner 5	−34.79	-1.07	0.28	
Scanner 6	−21.71	-0.7	0.48	

Note: A linear regression was used for this analysis (n= 703 participants). Age was mean centered, sex was effect-coded (female = -1, male = 1), scanner was dummy coded, and family income (in thousands of dollars) was log transformed prior to being included in the model. FDR corrected *p* value is reported in brackets for the critical effect of interest.

Supplementary Table 7. Family income and hippocampal sub-region interaction

Variable	*b*	*t*	*p*	*Marginal R^2*
Income	43.217	3.786	<0.001***	0.1
Region	602.522	3.356	<0.001***	
Age	14.924	8.655	<0.001***	
Sex	13.833	1.779	0.07547	
Scanner 1	-99.373	-3.391	0.01*	
Scanner 2	-51.182	-1.786	0.0743	
Scanner 3	-78.083	-2.631	0.008*	
Scanner 4	-26.876	-0.966	0.33	
Scanner 5	-74.509	-2.207	0.028*	
Scanner 6	-144.218	-5.205	<0.001**	
Region * Income	-58.118	-3.626	<0.001*** (<0.001***)	

Note: A linear mixed effects interaction model was used for this analysis (n= 703 participants). Volumes were modelled as a function of region, income, and their interaction. Because regions were nested within participants, we modelled a random intercept for participants, i.e., *Volumes ~ region * income + age + sex + scanner + (1|participant)*. Prior to running the model, age was mean centered, and sex was effect-coded (female = -1, male = 1), scanner and region were dummy coded (anterior = 0, posterior = 1) and income (in thousands of dollars) was log transformed. FDR corrected *p* value is reported in brackets for the critical effect of interest.

Supplementary Table 8. Anterior hippocampal volumes and memory relationship

Variable	*b*	*t*	*p*	*Adjusted R^2*
				0.49
Anterior volume	0.003	2.79	0.005** (0.02*)	
Age	1.52	24.82	<0.001***	
Sex	−0.11	−0.36	0.72	

Note: A linear regression was used for this analysis (n= 690 participants). Age was mean centered and sex was effect-coded (female = -1, male = 1) prior to being included in the analysis. FDR corrected *p* value is reported in brackets for the critical effect of interest.

Supplementary Table 9. Anterior hippocampal volumes and vocabulary relationship

Variable	*b*	*t*	*p*	*Adjusted R^2*
				0.63
Anterior volume	0.0004	3.66	<0.001*** (0.002**)	
Age	0.22	33.1	<0.001***	

Sex	0.08	2.59	0.010*	

Note: A linear regression was used for this analysis (n= 690 participants). Age was mean centered and sex was effect-coded (female = -1, male = 1) prior to being included in the analysis. FDR corrected *p* value is reported in brackets for the critical effect of interest.

Supplementary Table 10. Posterior hippocampal volumes and memory relationship

Variable	*b*	*t*	*p*	*Adjusted R^2*
				0.48
Posterior volume	0.002	0.22	0.828 (0.83)	
Age	1.54	24.1	<0.001***	
Sex	-0.01	-0.04	0.97	

Note: A linear regression was used for this analysis (n= 690 participants). Age was mean centered and sex was effect-coded (female = -1, male = 1) prior to being included in the analysis. FDR corrected *p* value is reported in brackets for the critical effect of interest.

Supplementary Table 11. Posterior hippocampal volumes and vocabulary relationship

Variable	*b*	*t*	*p*	*Adjusted R^2*
				0.664
Posterior volume	0.006	0.52	0.606 (0.72)	
Age	0.22	31.93	<0.001***	
Sex	0.1	3	0.003**	

Note: A linear regression was used for this analysis (n= 690 participants). Age was mean centered and sex was effect-coded (female = -1, male = 1) prior to being included in the analysis. FDR corrected *p* value is reported in brackets for the critical effect of interest.

Supplementary Table 12. Income subsample moderation of income-memory relations

Variable	*b*	*t*	*p*	*Adjusted R^2*
				0.51
Income	0.00004	4.9	<0.001***	
Income subsample	1.14	1.47	0.143	
Age	1.58	26.31	<0.001***	
Sex	0.06	0.19	0.85	

Income * Income subsample	−0.00002	−3.03	0.003** (0.007**)	

Note: A linear regression interaction model was used for this analysis (n= 690 participants). Age was mean centered, sex and income subsamples were effect-coded (female = -1, male = 1; income ≤$75k = -1, income >$75k = 1*)* before being included in the analysis. FDR corrected *p* value is reported in brackets for the critical effect of interest.

Summary of findings presented in Supplementary Table 12: The significant interaction between income and income subsample indicates that the positive relationship between income and episodic memory scores was stronger in the lower as compared to higher income subsample.

Supplementary Table 13. Income subsample moderation of income-vocabulary relations

Variable	*b*	*t*	*p*	*Adjusted R^2*
				0.51
Income	0.000005	6.22	<0.001***	
Income subsample	0.23	2.87	0.004**	
Age	0.22	36.15	<0.001***	
Sex	0.11	3.56	<0.001***	
Income * Income subsample	−0.000003	−3.64	<0.001*** (0.002**)	

Note: A linear regression interaction model was used for this analysis (n= 690 participants). Age was mean centered, sex and income subsamples were effect-coded (female = -1, male = 1; income ≤$75k = -1, income >$75k = 1) before being included in the analysis. FDR corrected *p* value is reported in brackets for the critical effect of interest.

Summary of findings presented in Supplementary Table 13: The significant interaction between income and income subsample indicates that the positive relationship between income and vocabulary scores was stronger in the lower as compared to higher income subsample.

Supplementary Table 14. Income group moderation of income-anterior volume relations

Variable	*b*	*t*	*p*	*Adjusted R^2*
				0.11
Income	0.0008	2.6	0.009**	

Income subsample	65.01	2.09	0.037*
Age	11.51	4.43	<0.001**
Sex	33.33	2.86	0.004**
Scanner 1	−40.83	−1.41	0.161
Scanner 2	−59.34	−2.00	0.046*
Scanner 3	13.1	0.46	0.646
Scanner 4	81.47	3.02	0.003**
Scanner 5	19.08	0.53	0.598
Scanner 6	−136.47	−4.86	<0.001***
Income * Income subsample	−0.0008	−2.57	0.01* (0.07)

Note: A linear regression interaction model was used for this analysis (n= 703 participants). Age was mean centered, sex and income subsamples were effect-coded (female = -1, male = 1; income ≤$75k = -1, income >$75k = 1) before being included in the analysis. FDR corrected *p* value is reported in brackets for the critical effect of interest.

Summary of findings presented in Supplementary Table 14: The significant interaction between income and income subsample indicates that the positive relationship between income and anterior hippocampal volumes was stronger in the lower as compared to higher income subsample.

Supplementary Table 15. Income group moderation of income-posterior volume relation

Variable	*b*	*t*	*p*	*Adjusted R^2*
				0.11
Income	−0.0005	-1.93	0.054	
Income subsample	-31.36	-1.18	0.24	
Age	18.19	8.14	< 0.001***	
Sex	-5.69	-0.57	0.57	
Scanner 1	-22.12	-0.89	0.38	
Scanner 2	37.85	1.48	0.14	
Scanner 3	17.99	0.74	0.46	
Scanner 4	0.94	0.04	0.97	
Scanner 5	-30.52	-0.98	0.33	
Scanner 6	-16.72	-0.69	0.49	
Income * Income subsample	0.0006	2.06	0.04* (0.11)	

Note: A linear regression interaction model was used for this analysis (n= 703 participants). Age was mean centered, sex and income subsamples were effect-coded (female = -1, male = 1; income ≤$75k = -1, income >$75k = 1) before being included in

the analysis. FDR corrected *p* value is reported in brackets for the critical effect of interest.

Summary of findings presented in Supplementary Table 15: The significant interaction between income and income subsample indicates that the negative relationship between income and posterior hippocampal volumes was stronger in the lower as compared to higher income subsample.

Supplementary Table 16. Income group moderation of anterior volume-memory relation

Variable	*b*	*t*	*p*	*Adjusted R^2*
				0.496
Anterior volumes	0.002	2.51	0.012*	
Income subsample	3.16	1.42	0.155	
Age	1.52	24.94	<0.001***	
Sex	−0.10	−0.33	0.738	
Anterior volumes * Income subsample	−0.001	−1.09	0.275 (0.62)	

Note: A linear regression interaction model was used for this analysis (n= 690 participants). Age was mean centered, sex and income subsamples were effect-coded (female = -1, male = 1; income ≤$75k = -1, income >$75k = 1) before being included in the analysis. FDR corrected *p* value is reported in brackets for the critical effect of interest.

Summary of findings presented in Supplementary Table 16: Here, we found a non-significant interaction between anterior hippocampal volumes and income subsample in predicting memory scores. This indicates that the positive relationship between anterior hippocampal volumes and memory scores does not differ in the lower (≤75k) and higher (>75k) subsample.

Supplementary Table 17. Income moderation of anterior volume-vocabulary relation

Variable	*b*	*t*	*p*	*Adjusted R^2*
				0.653
Ahipp vol	0.0003	3.44	<0.001***	
Income subsample	0.08	0.36	0.72	
Age	0.22	34.15	<0.001***	
Sex	0.09	2.93	0.004**	
Ahipp volume * Income subsample	0.00005	0.51	0.611 (0.75)	

Note: A linear regression interaction model was used for this analysis (n= 690 participants). Age was mean centered, sex and income subsample were effect-coded (female = -1, male = 1; income ≤$75k = -1, income >$75k = 1) before being included in the analysis. FDR corrected *p* value is reported in brackets for the critical effect of interest.

Summary of findings presented in Supplementary Table 17: Here, we found a non-significant interaction between anterior hippocampal volumes and income subsample in predicting vocabulary scores. This indicates that the positive relationship between anterior hippocampal volumes and vocabulary scores does not differ in the lower (≤75k) and higher (>75k) subsample.

Supplementary Table 18. Income moderation of posterior volume-memory relation

Variable	*b*	*t*	*p*	*Adjusted R^2*
				0.49
Phipp vol	0.0001	0.13	0.89	
Income subsample	2.844	1.13	0.26	
Age	1.543	24.13	<0.001***	
Sex	0.019	0.06	0.95	
Phipp volume * Income subsample	-0.001	-0.82	0.41 (0.66)	

Note: A linear regression interaction model was used for this analysis (n= 690 participants). Age was mean centered, sex and income subsample were effect-coded (female = -1, male = 1; income ≤$75k = -1, income >$75k = 1) before being included in the analysis. FDR corrected *p* value is reported in brackets for the critical effect of interest.

Summary of findings presented in Supplementary Table 18: Here, we found a non-significant interaction between posterior hippocampal volumes and income subsample in predicting memory scores. This indicates that the relationship between posterior hippocampal volumes and memory scores does not differ in the lower (≤75k) and higher (>75k) subsample.

Supplementary Table 19. Income moderation of posterior volume-vocabulary relation

Variable	*b*	*t*	*p*	*Adjusted R^2*
				0.648
Phipp vol	0.00006	0.52	0.61	
Income subsample	0.21	0.82	0.41	
Age	0.22	32.86	<0.001***	
Sex	0.1	3.29	0.001	
Phipp volume * Income subsample	−0.000004	-0.04	0.97 (0.97)	

Note: A linear regression interaction model was used for this analysis (n= 690 participants). Age was mean centered, sex and income subsample were effect-coded (female = -1, male = 1; income ≤$75k = -1, income >$75k = 1) before being included in

the analysis. FDR corrected *p* value is reported in brackets for the critical effect of interest.

Summary of findings presented in Supplementary Table 19: Here, we found a non-significant interaction between posterior hippocampal volumes and income subsample in predicting vocabulary scores. This indicates that the relationship between posterior hippocampal volumes and vocabulary scores does not differ in the lower (≤75k) and higher (>75k) subsample.

Supplementary Table 20. Income and memory relationship in lower income subsample

Variable	*b*	*t*	*p*	*Adjusted R^2*
				0.51
Income	2.25	4.66	<0.001*** (<0.001)	
Age	1.61	20.45	<0.001***	
Sex	0.21	0.53	0.593	

Note: A linear regression was used for this analysis (n= 399 participants). Sex was effect-coded (female = -1, male = 1), age was mean centered, and income was log transformed in the model. FDR corrected *p* value is reported in brackets for the critical effect of interest.

Summary of findings presented in Supplementary Table 20: The significant relationship between income and episodic memory scores indicate that income positively correlates with episodic memory scores in the lower income subsample (≤75k).

Supplementary Table 21. Income and memory relations in higher income subsample

Variable	*b*	*t*	*p*	*Adjusted R^2*
				0.49
Income	2.85	2.04	0.043* (0.07)	
Age	1.53	16.47	<0.001**	
Sex	−0.22	−0.48	0.63	

Note: A linear regression was used for this analysis (n= 291 participants). Sex was effect-coded (female = -1, male = 1), age was mean centered, and income was log transformed in the model. FDR corrected *p* value is reported in brackets for the critical effect of interest.

Summary of findings presented in Supplementary Table 21: The significant relationship between income and episodic memory scores indicate that income positively correlates with episodic memory scores in the higher income subsample (>75k).

Supplementary Table 22. Income and vocabulary relations in lower income subsample

Variable	*b*	*t*	*p*	*Adjusted R^2*

				0.64
Income	0.25	4.85	<0.001*** (<0.001***)	
Age	0.22	26.57	<0.001***	
Sex	0.05	1.31	0.192	

Note: A linear regression was used for this analysis (n= 399 participants). Sex was effect-coded (female = -1, male = 1), and age was mean centered in the model. FDR corrected *p* value is reported in brackets for the critical effect of interest.

Summary of findings presented in Supplementary Table 22: The significant relationship between income and vocabulary scores indicate that income positively correlates with vocabulary scores in the lower income subsample (≤75k).

Supplementary Table 23. Income-vocabulary relationship in higher income subsample

Variable	*b*	*t*	*p*	*Adjusted R^2*
				0.688
Income	0.44	3.18	0.002**(0.005*)	
Age	0.23	24.73	<0.001***	
Sex	0.18	4.06	<0.001***	

Note: A linear regression was used for this analysis (n= 291 participants). Sex was effect-coded (female = -1, male = 1), age was mean centered, and income was log transformed in the model. FDR corrected *p* value is reported in brackets for the critical effect of interest.

Summary of findings presented in Supplementary Table 23: The significant relationship between income and vocabulary scores indicate that income positively correlates with vocabulary scores in the higher income subsample (>75k).

Supplementary Table 24. Income-anterior volume relations in lower income subsample

Variable	*b*	*t*	*p*	*Adjusted R^2*
				0.12
Income	62.08	3.31	0.001** (0.01*)	
Age	12.3	3.67	<0.001***	
Sex	45.56	3.02	0.003**	
Scanner 1	−61.64	−1.67	0.096	
Scanner 2	−29.74	−0.73	0.464	
Scanner 3	3.94	0.11	0.911	
Scanner 4	116.99	3.41	<0.001***	
Scanner 5	−0.76	−0.02	0.988	
Scanner 6	−119.88	−3.27	<0.001***	

Note: A linear regression was used for this analysis (n= 410 participants). Age was mean centered, sex was effect-coded (female = -1, male = 1), income was log transformed, scanner was dummy coded prior to being included in the model. FDR corrected *p* value is reported in brackets for the critical effect of interest.

Summary of findings presented in Supplementary Table 24: The significant relationship between income and anterior hippocampal volumes indicate that income positively correlates with anterior hippocampal volumes in the lower income subsample (≤75k).

Supplementary Table 25. Income-anterior volume relations in higher income subsample

Variable	*b*	*t*	*p*	*Adjusted R^2*
				0.1
Income	−4.12	−0.07	0.94 (0.944)	
Age	8.49	2.03	0.044*	
Sex	11.03	0.59	0.556	
Scanner 1	−10.60	−0.23	0.822	
Scanner 2	−89.99	−2.06	0.04*	
Scanner 3	47.57	0.99	0.324	
Scanner 4	12.62	0.29	0.773	
Scanner 5	39.59	0.72	0.47	
Scanner 6	−165.63	−3.80	0.001**	

Note: A linear regression was used for this analysis (n= 291 participants). Age was mean centered, sex was effect-coded (female = -1, male = 1), income was log transformed, scanner was dummy coded prior to being included in the model. FDR corrected *p* value is reported in brackets for the critical effect of interest.

Summary of findings presented in Supplementary Table 25: The non-significant relationship between income and anterior hippocampal volumes indicate that income does not correlate with anterior hippocampal volumes in the higher income subsample (>75k).

Supplementary Table 26. Income-posterior volume relations in lower income subsample

Variable	*b*	*t*	*p*	*Adjusted R^2*
				0.06
Income	-35.23	-2.2	0.03* (0.09)	
Age	19.29	6.76	<0.001**	
Sex	-17.1	-1.33	0.18	
Scanner 1	-34.26	-1.09	0.28	
Scanner 2	27.39	0.79	0.43	
Scanner 3	15.5	0.52	0.61	
Scanner 4	7.7	0.26	0.79	
Scanner 5	17.92	0.44	0.66	
Scanner 6	-24.02	-0.77	0.44	

Note: A linear regression was used for this analysis (n= 410 participants). Age was mean centered, sex was effect-coded (female = -1, male = 1), income was log transformed, and scanner was dummy coded prior to being included in the model. FDR corrected *p* value is reported in brackets for the critical effect of interest.

Summary of findings presented in Supplementary Table 26: The significant relationship between income and posterior hippocampal volumes indicate that income negatively correlates with posterior hippocampal volumes in the lower income subsample (≤75k).

Supplementary Table 27. Income-posterior volume relations in higher income group

Variable	*b*	*t*	*p*	*Adjusted R^2*
				0.15
Income	-4.42	-0.09	0.93 (0.94)	
Age	16.57	4.51	<0.001**	
Sex	7.87	0.48	0.63	
Scanner 1	-1.23	-0.03	0.98	
Scanner 2	49.28	1.28	0.2	
Scanner 3	12.66	0.3	0.76	
Scanner 4	-8.6	-0.22	0.82	
Scanner 5	-85.38	-1.78	0.08	
Scanner 6	-9.45	-0.25	0.81	

Note: A linear regression was used for this analysis (n= 293 participants). Age was mean centered, sex was effect-coded (female = -1, male = 1), income was log transformed, and scanner was dummy coded prior to being included in the model. FDR corrected *p* value is reported in brackets for the critical effect of interest.

Summary of findings presented in Supplementary Table 27: The non-significant relationship between income and posterior hippocampal volumes indicate that income does not correlate with posterior hippocampal volumes in the higher income subsample (>75k).

Supplementary Table 28. Anterior volume-memory relationship in lower income group

Variable	*b*	*t*	*p*	*Adjusted R^2*
				0.49
Anterior volume	0.003	2.63	0.009** (0.04*)	
Age	1.51	18.81	<0.001***	
Sex	0.009	0.02	0.98	

Note: A linear regression was used for this analysis (n= 399 participants). Age was mean centered and sex was effect-coded (female = -1, male = 1) prior to being included

in the analysis. FDR corrected *p* value is reported in brackets for the critical effect of interest.

Summary of findings presented in Supplementary Table 28: The significant relationship between anterior hippocampal volumes and episodic memory scores indicate that anterior hippocampal volumes positively correlate with episodic memory scores in the lower income subsample (≤ 75k).

Supplementary Table 29. Anterior volume-memory relationship in higher income group

Variable	*b*	*t*	*p*	*Adjusted R^2*
				0.49
Anterior volume	0.001	0.97	0.334 (0.66)	
Age	1.53	16.22	<0.001***	
Sex	−0.24	−0.53	0.598	

Note: A linear regression was used for this analysis (n= 291 participants). Age was mean centered and sex was effect-coded (female = -1, male = 1) prior to being included in the analysis. FDR corrected *p* value is reported in brackets for the critical effect of interest.

Summary of findings presented in Supplementary Table 29: The nonsignificant relationship between anterior hippocampal volumes and episodic memory scores indicate that anterior hippocampal volumes do not correlate with episodic memory scores in the higher income subsample (>75k).

Supplementary Table 30. Anterior volume-vocabulary relations in lower income group

Variable	*b*	*t*	*p*	*Adjusted R^2*
				0.62
Anterior volume	0.0003	2.45	0.015* (0.05*)	
Age	0.21	24.74	<0.001***	
Sex	0.03	0.79	0.43	

Note: A linear regression was used for this analysis (n= 399 participants). Age was mean centered and sex was effect-coded (female = -1, male = 1) prior to being included in the analysis. FDR corrected *p* value is reported in brackets for the critical effect of interest.

Summary of findings presented in Supplementary Table 30: The significant relationship between anterior hippocampal volumes and vocabulary scores indicate that anterior hippocampal volumes positively correlate with vocabulary scores in the lower income subsample (≤75k).

Supplementary Table 31. Anterior volume-vocabulary relations in higher income group

Variable	*b*	*t*	*p*	*Adjusted R^2*
				0.64
Anterior volume	0.0004	2.58	0.011* (0.044*)	
Age	0.23	24.17	<0.001***	
Sex	0.17	3.89	<0.001***	

Note: A linear regression was used for this analysis (n= 291 participants). Age was mean centered and sex was effect-coded (female = -1, male = 1) prior to being included in the analysis. FDR corrected *p* value is reported in brackets for the critical effect of interest.

Summary of findings presented in Supplementary Table 31: The significant relationship between anterior hippocampal volumes and vocabulary scores indicate that anterior hippocampal volumes positively correlate with vocabulary scores in the higher income subsample (>$75k).

Supplementary Table 32. Posterior volume-memory relations in the lower income group

Variable	*b*	*t*	*p*	*Adjusted R^2*
				0.49
Posterior volume	0.001	0.81	0.41 (.66)	
Age	1.53	17.8	<0.001***	
Sex	0.23	5.78	0.56	

Note: A linear regression was used for this analysis (n= 399 participants). Age was mean centered and sex was effect-coded (female = -1, male = 1) prior to being included in the analysis. FDR corrected *p* value is reported in brackets for the critical effect of interest.

Summary of findings presented in Supplementary Table 32: The nonsignificant relationship between posterior hippocampal volumes and episodic memory scores indicate that posterior hippocampal volumes do not correlate with episodic memory scores in the lower income subsample (≤ 75k).

Supplementary Table 33. Posterior volume-memory relations in higher income group

Variable	*b*	*t*	*p*	*Adjusted R^2*
				0.49
Posterior volume	−0.0008	-0.52	0.60 (0.75)	

Age	1.56	16.13	<0.001**	
Sex	−0.21	-0.47	0.63	

Note: A linear regression was used for this analysis (n= 291 participants). Age was mean centered and sex was effect-coded (female = -1, male = 1) prior to being included in the analysis. FDR corrected *p* value is reported in brackets for the critical effect of interest.

Summary of findings presented in Supplementary Table 33: The nonsignificant relationship between posterior hippocampal volumes and episodic memory scores indicate that posterior hippocampal volumes do not correlate with episodic memory scores in the higher income subsample (>75k).

Supplementary Table 34. Posterior volume-vocabulary relations in lower income group

Variable	*b*	*t*	*p*	*Adjusted R^2*
				0.64
Posterior volume	0.0001	0.59	0.55 (0.75)	
Age	0.23	23.43	<0.001***	
Sex	0.06	1.32	0.19	

Note: A linear regression was used for this analysis (n= 399 participants). Age was mean centered and sex was effect-coded (female = -1, male = 1) prior to being included in the analysis. FDR corrected *p* value is reported in brackets for the critical effect of interest.

Summary of findings presented in Supplementary Table 34: The nonsignificant relationship between posterior hippocampal volumes and vocabulary scores indicate that posterior hippocampal volumes do not correlate with vocabulary scores in the lower income subsample (≤75k).

Supplementary Table 35. Posterior volume-vocabulary relations in higher income group

Variable	*b*	*t*	*p*	*Adjusted R^2*
				0.68
Posterior volume	−0.00001	-0.044	0.96 (0.97)	
Age	0.23	23.76	<0.001***	
Sex	0.18	3.97	<0.001***	

Note: A linear regression was used for this analysis (n= 291 participants). Age was mean centered and sex was effect-coded (female = -1, male = 1) prior to being included in the analysis. FDR corrected *p* value is reported in brackets for the critical effect of interest.

Summary of findings presented in Supplementary Table 35: The nonsignificant relationship between posterior hippocampal volumes and vocabulary scores indicate

that posterior hippocampal volumes do not correlate with vocabulary scores in the higher income subsample (>75k).

Supplementary Table 36. Age moderation of income-memory score relationship

Variable	*b*	*t*	*p*	*Adjusted R^2*
Age	1.03	1.52	0.13	0.51
Income	1.61	5.08	<0.001***	
Sex	0.06	0.19	0.848	
Age * Income	0.05	0.81	0.420 (0.51)	

Note: A linear regression interaction model was used for this analysis (n= 690 participants). Age was mean centered, income was log transformed, and sex was effect-coded (female = -1, male = 1) before being included in the analysis. Episodic memory scores were missing from 13 of the 703 participants included in the volume analyses. FDR corrected *p* value is reported in brackets for the critical effect of interest.

Summary of findings presented in Supplementary Table 36: Here, we found a nonsignificant interaction between age and income to predict memory scores. This indicates that age does not moderate the positive relationship between income and memory scores.

Supplementary Table 37. Age moderation of income-vocabulary score relationship

Variable	*b*	*t*	*p*	*Adjusted R^2*
Age	0.16	2.27	0.023*	0.66
Income	0.28	8.39	<0.001***	
Sex	0.11	3.53	<0.001***	
Age * Income	0.006	0.93	0.354 (0.46)	

Note: A linear regression interaction model was used for this analysis (n= 690 participants). Age was mean centered, income was log transformed, and sex was effect-coded (female = -1, male = 1) before being included in the analysis. Vocabulary scores were missing from 13 of the 703 participants included in the volume analyses. FDR corrected *p* value is reported in brackets for the critical effect of interest.

Summary of findings presented in Supplementary Table 37: Here, we found a nonsignificant interaction between age and income to predict vocabulary scores. This indicates that age does not moderate the positive relationship between income and vocabulary scores.

Supplementary Table 38. Age moderation of income-anterior volume relationship

Variable	*b*	*t*	*P*	*Adjusted R^2*
Age	28.16	1.07	0.284	0.11
Income	44.74	3.6	<0.001***	
Sex	33	2.83	0.005**	
Scanner 1	−37.08	−1.28	0.202	
Scanner 2	−62.82	−2.12	0.035*	
Scanner 3	14.85	0.52	0.602	
Scanner 4	79.38	2.94	0.003**	
Scanner 5	21.09	0.58	0.56	
Scanner 6	−136.38	−4.86	<0.001***	
Age * Income	−1.52	−0.64	0.519 (0.61)	

Note: A linear regression interaction model was used for this analysis (n= 703 participants). Age was mean centered, income was log transformed, sex was effect-coded (female = -1, male = 1) and scanner was dummy coded before being included in the analysis. FDR corrected *p* value is reported in brackets for the critical effect of interest.

Summary of findings presented in Supplementary Table 38: Here, we found a nonsignificant interaction between age and income to predict anterior hippocampal volumes. This indicates that age does not moderate the relationship between income and anterior hippocampal volumes.

Supplementary Table 39. Age moderation of income-posterior volume relationship

Variable	*b*	*t*	*p*	*Adjusted R^2*
Age	33.69	1.49	0.14	0.11
Income	-13.49	-1.26	0.21	
Sex	-5.63	-0.56	0.58	
Scanner 1	-24.21	-0.97	0.33	
Scanner 2	39.63	1.55	0.12	
Scanner 3	20.06	0.82	0.41	
Scanner 4	-0.74	-0.03	0.98	
Scanner 5	-32.29	-1.04	0.3	
Scanner 6	-17.39	-0.72	0.47	
Age * Income	-1.36	-0.67	0.50 (0.60)	

Note: A linear regression interaction model was used for this analysis (n= 703 participants). Age was mean centered, income was log transformed, sex was effect-coded (female = -1, male = 1) and scanner was dummy coded before being included in the analysis. FDR corrected *p* value is reported in brackets for the critical effect of interest.

Summary of findings presented in Supplementary Table 39: Here, we found a nonsignificant interaction between age and income to predict posterior hippocampal volumes. This indicates that age does not moderate the relationship between income and posterior hippocampal volumes.

Supplementary Table 40. Age moderation of anterior volume-memory relationship

Variable	*b*	*t*	*p*	*Adjusted R^2*
Age	2.1	4.81	<.001***	0.49
Anterior volume	0.003	2.88	0.004*	
Sex	-0.12	-0.39	0.69	
Age * Anterior volume	-0.0002	-1.34	0.18 (0.47)	

Note: A linear regression interaction model was used for this analysis (n= 690 participants). Age was mean centered and sex was effect-coded (female = -1, male = 1). FDR corrected *p* value is reported in brackets for the critical effect of interest.

Summary of findings presented in Supplementary Table 40: Here, we found a nonsignificant interaction between age and anterior hippocampal volumes to predict episodic memory scores. This indicates that age does not moderate the relationship between anterior hippocampal volumes and episodic memory scores.

Supplementary Table 41. Age moderation of anterior volume-vocabulary relationship

Variable	*b*	*t*	*p*	*Adjusted R^2*
Age	0.23	5.27	<.001***	0.67
Anterior volume	0	3.02	.003**	
Sex	0.1	3.13	.002**	
Age * Anterior volume	0	-0.27	0.789 (0.90)	

Note: A linear regression interaction model was used for this analysis (n= 690 participants). Age was mean centered and sex was effect-coded (female = -1, male = 1). FDR corrected *p* value is reported in brackets for the critical effect of interest.

Summary of findings presented in Supplementary Table 41: Here, we found a nonsignificant interaction between age and anterior hippocampal volumes to predict vocabulary scores. This indicates that age does not moderate the relationship between anterior hippocampal volumes and vocabulary scores.

Supplementary Table 42. Age moderation of posterior volume-memory relationship

Variable	*b*	*t*	*p*	*Adjusted R^2*
Age	3.05	5.98	<0.001***	0.51
Posterior volume	0.0005	0.47	0.64	
Sex	-0.1	-0.32	0.74	
Age * Posterior volume	-0.001	-2.98	0.003** (0.04*)	

Note: A linear regression interaction model was used for this analysis (n= 690 participants). Age was mean centered and sex was effect-coded (female = -1, male = 1) before being included in the analysis. FDR corrected *p* value is reported in brackets for the critical effect of interest.

Summary of findings presented in Supplementary Table 42: Here, we found a significant interaction between age and posterior hippocampal volumes to predict episodic memory scores. This indicates that age moderates the relationship between posterior hippocampal volumes and episodic memory scores.

Supplementary Table 43. Age moderation of posterior volume-vocabulary relationship

Variable	*b*	*t*	*p*	*Adjusted R^2*
Age	0.37	6.83	<0.001***	0.67
Posterior volume	0.00009	0.76	0.45	
Sex	0.09	2.73	0.006**	
Age * Posterior volume	0.00007	-2.85	0.005** (0.04*)	

Note: A linear regression interaction model was used for this analysis (n= 690 participants). Age was mean centered and sex was effect-coded (female = -1, male = 1) before being included in the analysis. FDR corrected *p* value is reported in brackets for the critical effect of interest.

Summary of findings presented in Supplementary Table 43: Here, we found a significant interaction between age and posterior hippocampal volumes to predict vocabulary scores. This indicates that age moderates the relationship between posterior hippocampal volumes and vocabulary scores.

Supplementary Table 44. Parental education and memory score relationship

Variable	*b*	*t*	*p*	*Adjusted R^2*
				0.5
Parental education	0.4	3.02	0.003** (0.01*)	
Age	1.59	26.07	<0.001***	

Sex	−0.008	−0.03	0.978	

Note: A linear regression model was used for this analysis (n= 679 participants). Sex was effect-coded (female = -1, male = 1), and age was mean centered in the model. Cognitive scores were missing for 10 of the 689 participants who were included in the education analyses. FDR corrected *p* value is reported in brackets for the critical effect of interest.

Summary of findings presented in Supplementary Table 44: The significant relationship between parental education and episodic memory scores indicates that more years of parental education predicts better episodic memory scores.

Supplementary Table 45. Parental education and vocabulary score relationship

Variable	*b*	*t*	*p*	*Adjusted R^2*
				0.66
Parental education	0.11	8.24	<0.001*** (0.007**)	
Age	0.23	36.34	<0.001***	
Sex	0.09	3.11	0.002**	

Note: A linear regression model was used for this analysis (n= 679 participants). Sex was effect-coded (female = -1, male = 1), and age was mean centered in the model. Cognitive scores were missing for 10 of the 689 participants who were included in the education analyses. FDR corrected *p* value is reported in brackets for the critical effect of interest.

Summary of findings presented in Supplementary Table 45: The significant relationship between parental education and vocabulary scores indicates that more years of parental education predicts better vocabulary scores.

Supplementary Table 46. Parental education and anterior hippocampal volume relations

Variable	*b*	*t*	*p*	*Adjusted R^2*
				0.102
Parental education	12	2.26	0.024* (0.06)	
Age	10.76	4.08	<0.001***	
Sex	29.81	2.53	0.012*	
Scanner 1	−35.92	−1.22	0.221	
Scanner 2	−56.99	−1.90	0.058	
Scanner 3	10.88	0.38	0.703	
Scanner 4	83.17	3.02	0.003**	
Scanner 5	15.9	0.43	0.665	
Scanner 6	−140.35	−4.95	<0.001***	

Note: A linear regression model was used for this analysis and (n= 689 participants). Age was mean centered, sex was effect-coded (female = -1, male = 1), scanner was dummy coded prior to being included in the model. FDR corrected *p* value is reported in brackets for the critical effect of interest.

Summary of findings presented in Supplementary Table 46: The significant relationship between parental education and anterior hippocampal volumes indicates that more years of parental education predicts larger anterior hippocampal volumes.

Supplementary Table 47. Parental education and posterior hippocampal volume relation

Variable	*b*	*t*	*p*	*Adjusted R^2*
				0.127
Parental education	-13.281	-2.92	0.004** (.02*)	
Age	18.285	8.13	<0.001***	
Sex	-6.152	-0.61	0.54	
Scanner 1	-23.989	-0.96	0.34	
Scanner 2	43.575	1.7	0.09	
Scanner 3	14.509	0.6	0.55	
Scanner 4	-0.126	-0.01	1	
Scanner 5	-26.431	-0.84	0.4	
Scanner 6	-18.476	-0.76	0.45	

Note: A linear regression model was used for this analysis (n= 689 participants). Age was mean centered, sex was effect-coded (female = -1, male = 1), scanner was dummy coded prior to being included in the model. FDR corrected *p* value is reported in brackets for the critical effect of interest.

Summary of findings presented in Supplementary Table 47. The significant relationship between parental education and posterior hippocampal volumes indicates that more years of parental education predicts smaller posterior hippocampal volumes.

Supplementary Table 48. Minority status moderation of income-memory relationship

Variable	*b*	*t*	*p*	*adjusted R^2*
Income	2.200	4.420	<0.001***	0.520
Minority status	−6.52	−1.26	0.209	
Age	1.650	20.520	<0.001***	
Sex	0.110	0.280	0.777	
Income x Minority status	0.560	1.140	0.26 (0.40)	

Note: A linear regression interaction model was used for this analysis and included participants with low income subsample only (n= 391 participants). Age was mean centered, sex was effect-coded (female = -1, male = 1), income was log transformed

prior to being included in the model. FDR corrected *p* value is reported in brackets for the critical effect of interest.

Summary of findings presented in Supplementary Table 48: There was no significant interaction between minority status and income, indicating that income-episodic memory relationships were similar in minority status and non-minority status individuals. Notably, income remained significant even after accounting for minority status, highlighting the generalizability of income-episodic memory relationships.

Supplementary Table 49. Income-memory score relationship in low-income whites

Variable	*b*	*t*	*p*	*adjusted R^2*
Income	1.663	2.468	0.014* (0.03*)	0.530
Age	1.697	17.312	<0.001***	
Sex	-0.052	-0.108	0.914	

Note: A linear regression model was used for this analysis (n= 263 participants). Age was mean centered, sex was effect-coded (female = -1, male = 1) and income was log transformed prior to being included in the model. FDR corrected *p* value is reported in brackets for the critical effect of interest.

Summary of findings presented in Supplementary Table 49: The significant relationship between income and episodic memory scores in this model indicates that higher income correlates with better episodic memory scores in non-minority status individuals in the lower income subsample.

Supplementary Table 50. Income-memory score relationship in low-income non-whites

Variable	b	t	p	adjusted R^2
Income	2.683	3.591	<0.001*** (0.002**)	0.490
Age	1.559	11.056	<0.001***	
Sex	0.440	0.618	0.540	

Note: A linear regression model was used for this analysis (n= 128 participants). Age was mean centered, sex was effect-coded (female = -1, male = 1) and income was log transformed prior to being included in the model. FDR corrected *p* value is reported in brackets for the critical effect of interest.

Summary of findings presented in Supplementary Table 50: The significant relationship between income and episodic memory scores in this model indicates that higher income correlates with better episodic memory scores in minority status individuals in the lower income subsample.

Supplementary Table 51. Minority status moderation of income-vocabulary relationship

Variable	b	t	p	adjusted R^2
Income	0.200	3.810	<0.001***	0.660
Minority status	−0.32	−0.60	0.550	
Age	0.230	27.680	<0.001***	
Sex	0.030	0.640	0.525	
Income x Minority status	0.010	0.190	0.84 (0.85)	

Note: A linear regression interaction model was used for this analysis and included participants in the low income subsample only (n= 391 participants). Age was mean centered, sex was effect-coded (female = -1, male = 1) and income was log transformed prior to being included in the model. FDR corrected *p* value is reported in brackets for the critical effect of interest.

Summary of findings presented in Supplementary Table 51: There was no significant interaction between minority status and income to predict vocabulary scores, indicating that income-vocabulary relationships were similar in non-minority status and minority status individuals. The fact that income is still significant after accounting for minority status indicates highlights the generalizability of income-vocabulary relationships.

Supplementary Table 52. Income-vocabulary relationship in low-income non-whites

Variable	b	t	p	adjusted R^2
Income	0.200	3.042	0.003*** (0.007**)	0.720
Age	0.249	26.000	<0.001***	
Sex	0.008	0.166	0.868	

Note: A linear regression model was used for this analysis (n= 263 participants). Age was mean centered, sex was effect-coded (female = -1, male = 1) and income was log transformed prior to being included in the model. FDR corrected *p* value is reported in brackets for the critical effect of interest.

Summary of findings presented in Supplementary Table 52: The significant relationship between income and vocabulary scores in this model indicates that higher income correlates with better vocabulary scores in non-minority status individuals in the lower income subsample.

Supplementary Table 53. Income-vocabulary relationship in low-income non-whites

Variable	b	t	p	adjusted R^2
Income	0.170	2.093	0.038* (0.07)	0.540
Age	0.191	12.487	<0.001***	
Sex	0.060	0.774	0.441	

Note: A linear regression model was used for this analysis (n= 128 participants). Age was mean centered, sex was effect-coded (female = -1, male = 1) and income was log

transformed prior to being included in the model. FDR corrected *p* value is reported in brackets for the critical effect of interest.

Summary of findings presented in Supplementary Table 53: The significant relationship between income and vocabulary scores in this model indicates that higher income correlates with better vocabulary scores in minority status individuals in the lower income subsample.

Supplementary Table 54. Minority status moderation of income-anterior volume relation

Variable	b	t	p	Adjusted R^2
Income	49.85	2.63	0.009**	0.15
Minority status	−311.86	−1.60	0.111	
Age	14.11	4.22	0.001**	
Sex	39.25	2.61	0.009**	
Scanner 1	−32.15	−0.87	0.382	
Scanner 2	−46.80	−1.16	0.247	
Scanner 3	23.57	0.66	0.508	
Scanner 4	113.61	3.36	<0.001***	
Scanner 5	−25.22	−0.53	0.599	
Scanner 6	−117.13	−3.24	<0.001***	
Income x Minority status	23.72	1.28	0.201 (0.40)	

Note: A linear regression interaction model was used for this analysis and includeed individuals from the lower income subsample (n= 402 participants). Age was mean centered, sex was effect-coded (female = -1, male = 1), scanner was dummy coded and income was log transformed prior to being included in the model. FDR corrected *p* value is reported in brackets for the critical effect of interest.

Summary of findings presented in Supplementary Table 54: The non-significant interaction between income and minority status indicates that income-anterior hippocampal volume relationships were not significantly different in non-minority status and minority status individuals in the lower income subset of the sample. The fact that income remains significant after accounting for minority status in the model highlights the generalizability of the relationship between income and anterior hippocampal volumes.

Supplementary Table 55. Minority status moderation of income-posterior volume relation

Variable	b	t	p	Adjusted R^2
Income	-34.576	-2.09	0.04*	0.15
Minority status	140.981	-0.83	0.41	
Age	19.813	6.80	<0.001***	
Sex	-14.422	-1.10	0.27	
Scanner 1	-29.429	-0.92	0.36	

Scanner 2	34.000	0.97	0.33
Scanner 3	9.019	0.29	0.77
Scanner 4	4.734	0.16	0.87
Scanner 5	17.252	0.41	0.68
Scanner 6	-21.989	-0.70	0.49
Income x Minority status	13.367	0.83	0.41 (0.57)

Note: A linear regression interaction model was used for this analysis and included only individuals from the low income subsample (n= 402 participants). Age was mean centered, sex was effect-coded (female = -1, male = 1), scanner was dummy coded and income was log transformed prior to being included in the model. FDR corrected *p* value is reported in brackets for the critical effect of interest.

Summary of findings presented in Supplementary Table 55: The non-significant interaction between income and minority status indicates that income-posterior hippocampal volume relationships were not significantly different in non-minority status and minority status individuals in the lower income subset of the sample.

Supplementary Table 56. Income-anterior volume relationship in low income whites

Variable	b	t	p	Adjusted R^2
Income	23.414	0.897	0.37 (0.57)	0.150
Age	11.883	2.817	0.005**	
Sex	57.059	3.078	0.002**	
Scanner 1	20.080	0.404	0.686	
Scanner 2	-50.861	-1.100	0.273	
Scanner 3	-0.305	-0.006	0.995	
Scanner 4	110.688	2.736	0.007**	
Scanner 5	-42.349	-0.854	0.394	
Scanner 6	-153.684	-3.628	<0.001***	

Note: A linear regression model was used for this analysis (n= 268 participants). Age was mean centered, sex was effect-coded (female = -1, male = 1), scanner was dummy coded and income was log transformed prior to being included in the model. FDR corrected *p* value is reported in brackets for the critical effect of interest.

Summary of findings presented in Supplementary Table 56: The non-significant relationship between income and anterior hippocampal volumes indicates that higher income did not significantly correlate with anterior hippocampal volumes in non-minority status individuals in the lower income subset of the sample.

Supplementary Table 57. Income-anterior volume relationship in low income non-whites

Variable	b	t	p	Adjusted R^2
Income	66.441	2.396	0.018* (0.08)	0.100
Age	18.192	3.257	0.001**	
Sex	1.858	0.072	0.942	
Scanner 1	-125.527	-2.100	0.038	

Scanner 2	-84.926	-0.990	0.324
Scanner 3	12.019	0.207	0.836
Scanner 4	61.933	0.932	0.353
Scanner 5	216.220	1.196	0.234
Scanner 6	-50.393	-0.682	0.497

Note: A linear regression model was used for this analysis (n= 134 participants). Age was mean centered, sex was effect-coded (female = -1, male = 1), scanner was dummy coded and income was log transformed prior to being included in the model. FDR corrected *p* value is reported in brackets for the critical effect of interest.

Summary of findings presented in Supplementary Table 57: The significant relationship between income and anterior hippocampal volumes indicates that higher income significantly correlated with anterior hippocampal volumes in minority status individuals in the lower income subset of the sample.

Supplementary Table 58. Minority status moderation of age-anterior volume relationship

Variable	b	t	p	Adjusted R^2
Age	13.71	4.89	<0.001***	0.13
Minority status	−63.13	−4.32	<0.001***	
Income	28.93	2.3	0.022*	
Sex	28.59	2.46	0.014*	
Scanner 1	−16.62	−0.58	0.565	
Scanner 2	−74.26	−2.52	0.012*	
Scanner 3	28.49	1	0.319	
Scanner 4	78.63	2.96	0.003**	
Scanner 5	7.46	0.21	0.835	
Scanner 6	−134.44	−4.84	<0.001***	
Age x Minority status	2.42	0.92	0.356 (0.56)	

Note: A linear regression interaction model was used for this analysis (n= 695 participants). Age was mean centered, sex was effect-coded (female = -1, male = 1), scanner was dummy coded and income was log transformed prior to being included in the model. FDR corrected *p* value is reported in brackets for the critical effect of interest.

Summary of findings presented in Supplementary Table 58: The non-significant interaction between age and minority status indicates that the relationship between age and anterior hippocampal volumes is not significantly different in non-minority status and minority status individuals across the sample.

Supplementary Table 59. Minority moderation of age-anterior relation (low income)

Variable	b	t	p	Adjusted R^2
Age	14.275	4.136	<0.001***	0.15
Minority status	-63.326	3.696	<0.001***	
Income	48.951	2.577	0.010*	
Sex	39.426	2.617	0.009**	

Scanner 1	-31.785	0.863	0.389
Scanner 2	-46.337	1.146	0.253
Scanner 3	22.756	0.637	0.524
Scanner 4	116.255	3.439	<0.001***
Scanner 5	-26.664	0.555	0.579
Scanner 6	-115.271	3.184	0.002**
Age x minority status	0.666	0.212	0.832 (0.86)

Note: A linear regression interaction model was used for this analysis and only includeed individuals from the lower income subsample (n= 402 participants). Age was mean centered, sex was effect-coded (female = -1, male = 1), scanner was dummy coded and income was log transformed prior to being included in the model. FDR corrected *p* value is reported in brackets for the critical effect of interest.

Summary of findings presented in Supplementary Table 59: The non-significant interaction between age and minority status indicates that the relationship between age and anterior hippocampal volumes is not significantly different in non-minority status and minority status individuals in the lower income subset of the sample.

Supplementary Table 60. Age influences on subregion volumes

**Variable**	***b***	***t***	***p***
Age	11.23	4.81	**<0.001*** **
region	-46.09	−2.97	**0.003**
Scanner 1	-31.62	-1.63	**0.01** **
Scanner 2	-10.34	-0.52	0.60
Scanner 3	16.57	0.87	0.38
Scanner 4	40.87	2.27	**0.019***
Scanner 5	-6.76	-0.28	0.78
Scanner 6	-76.47	-4.08	**<0.001*** **
Gender	13.83	1.77	0.08
Income	14.16	1.74	0.08
age*region	7.40	2.36	**0.02* (0.04*)**

Note: Volumes were modelled as a function of region, age, sex, and the interaction between age and region. Because regions were nested within participants, we modelled a random intercept for participants to account for the random effect of participant on regional volumes, i.e., lmer(*Volumes ~ regions * age + sex + income + scanner + (1|participant)), n = 703.* Prior to running the model, age was mean centered, and volumes were dummy coded (anterior = 0), posterior = 1). FDR corrected *p* value is reported in brackets for the critical effect of interest.

Supplementary Figures

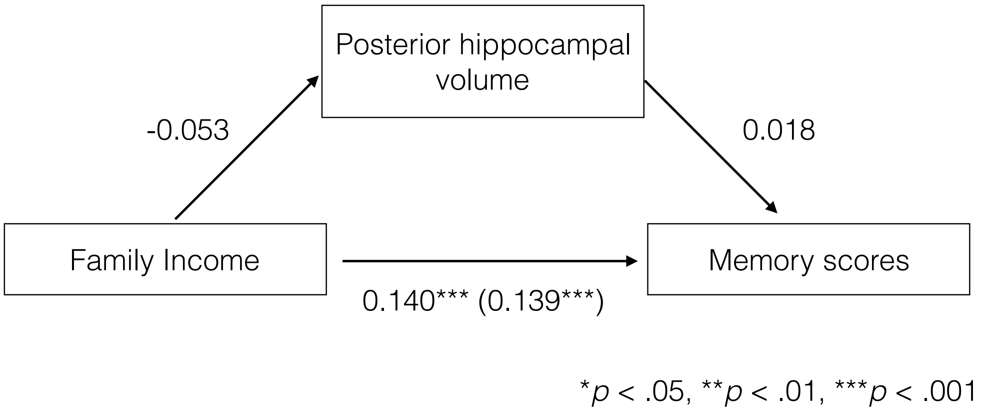

Supplementary Figure 1. Depiction of posterior hippocampal volume mediation analysis of income and memory score relationship. Standardized regression coefficients are reported. Bootstrapped mediation analyses (n=690 participants) revealed that the positive association between family income and memory scores was not mediated by posterior hippocampal volumes, *ab* = -0.001*, SE* = 0.002, 95% CI [-0.006, 0]. The total effect of family income on memory scores was significant, *c* = 0.139, *SE* = 0.29, 95% CI [0.083, 0.20], and the direct effect of family income on memory scores was not significantly altered when the indirect path *ab* through posterior hippocampal volumes was considered, *c'* = 0.140, *SE* = 0.029, 95% CI [0.083, 0.20]. Confidence intervals that do not include zero reflect an effect. False discovery rate adjusted p-values for the relationships displayed are presented in the main text.

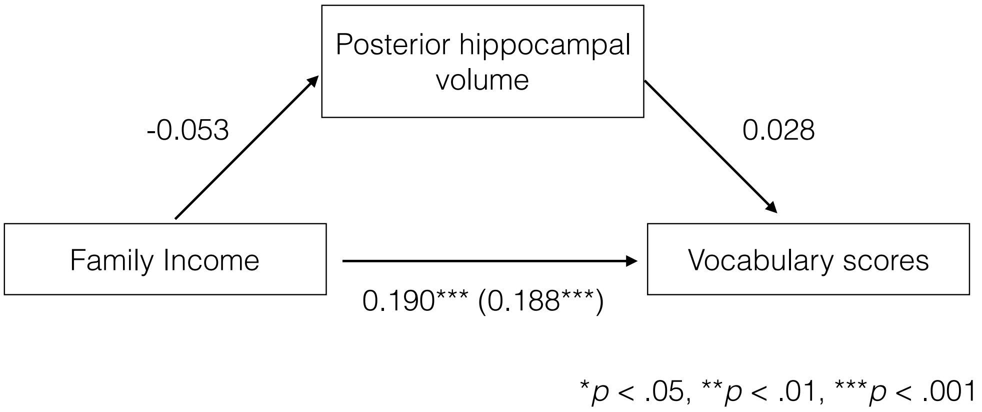

Supplementary Figure 2. Depiction of posterior hippocampal volume mediation analysis of income and vocabulary score relationship. Standardized regression coefficients are reported. Bootstrapped mediation analyses (n=690 participants) revealed that the positive association between family income and vocabulary scores was not mediated by posterior hippocampal volumes, *ab* = -0.001, *SE* = 0.002, 95% CI [-0.006, 0]. The total effect of family income on vocabulary scores was significant, *c* = 0.188, *SE* = 0.024, 95% CI [0.14, 0.24] and the direct effect of family income on vocabulary scores was not significantly altered when the indirect path *ab* through posterior hippocampal volumes was considered, *c'* = 0.190, *SE* = 0.025, 95% CI [0.140, 0.24]. Confidence intervals that do not include zero reflect an effect. False discovery rate adjusted p-values for the relationships displayed are presented in the main text.

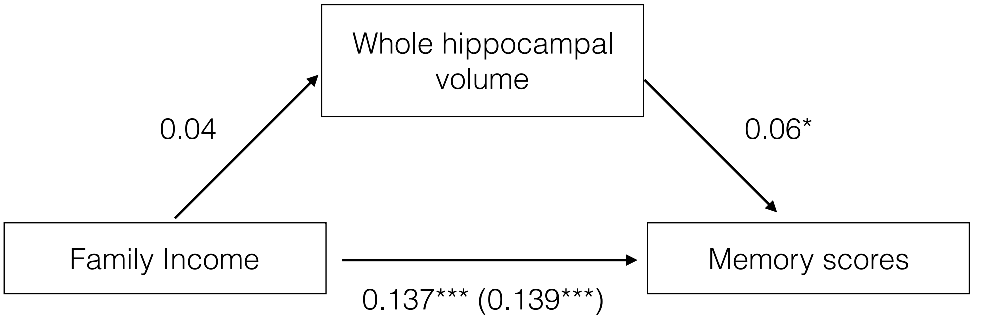

$^{*}p < .05$, $^{**}p < .01$, $^{***}p < .001$

Supplementary Figure 3. *Depiction of whole hippocampal volume mediation analysis of income and memory score relationship*. Standardized regression coefficients are reported. Bootstrapped mediation analyses revealed (n=690 participants) that the positive association between family income and memory scores was not mediated by whole hippocampal volumes, *ab* = 0.003, *SE* = 0.003, 95% CI [-0.002, 0.010]. The total effect of family income on memory scores was significant, *c* = 0.139, *SE* =0.028, 95% CI[0.082, 0.19], and the direct effect of family income on memory scores was not significantly altered when the indirect path *ab* through whole hippocampal volumes was considered, *c'* = 0.137, *SE* = 0.028, 95% CI [0.080, 0.19]. Confidence intervals that do not include zero reflect an effect. False discovery rate adjusted p-values for the relationships between income and memory scores displayed above can be found in the main text.

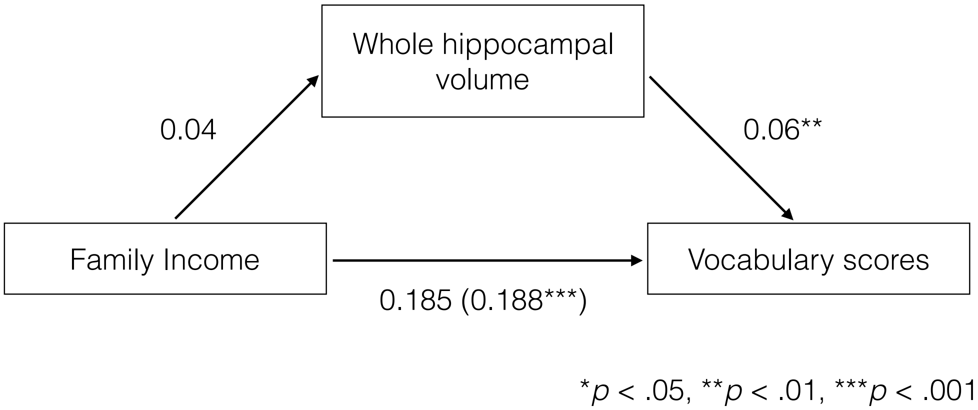

Supplementary Figure 4. Depiction of whole hippocampal volume mediation analysis of income and vocabulary score relationship. Standardized regression coefficients are reported. Bootstrapped mediation analyses (n=690 participants) revealed that the positive association between family income and vocabulary scores was not mediated by whole hippocampal volumes, *ab* = 0.003, *SE* =0.003, 95% CI [-0.002, 0.01]. The total effect of family income on vocabulary scores was significant, *c* = 0.188, *SE* = 0.024, 95% CI [0.14, 0.24], and the direct effect of family income and vocabulary scores were not significantly altered when the indirect path *ab* through whole hippocampal volumes was considered, *c'* = 0.185, *SE* = 0.024, 95% CI 0.14, 0.23]. Confidence intervals that do not include zero reflect an effect. False discovery rate adjusted p-values for the relationships between income and vocabulary scores displayed above can be found in the main text.

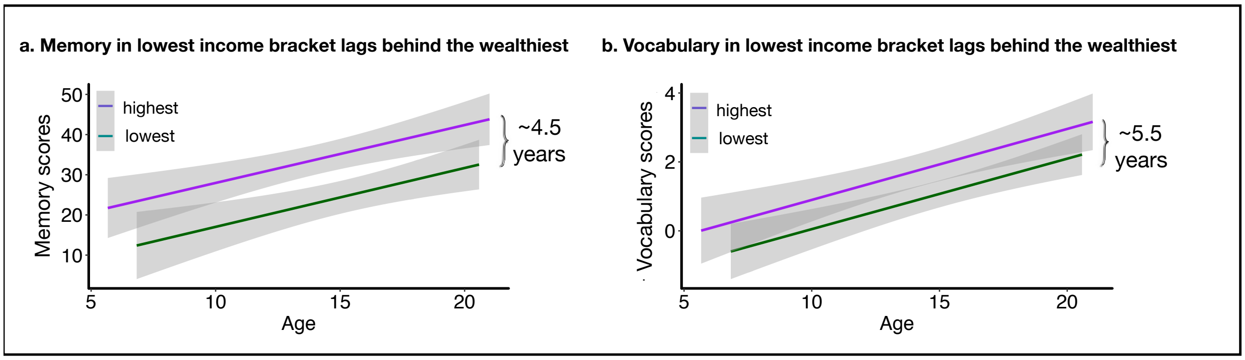

Supplementary Figure 5. Relationships between age and cognitive scores for the lowest and higher income bracket. Linear regression model estimates revealed that a) memory scores in the lowest income subsample (green) lagged behind the wealthiest (purple) by ~ 4.5 years, and b) vocabulary scores by ~ 5.5 years (n=46 participants). Grey shading reflects 95% confidence intervals around the mean.

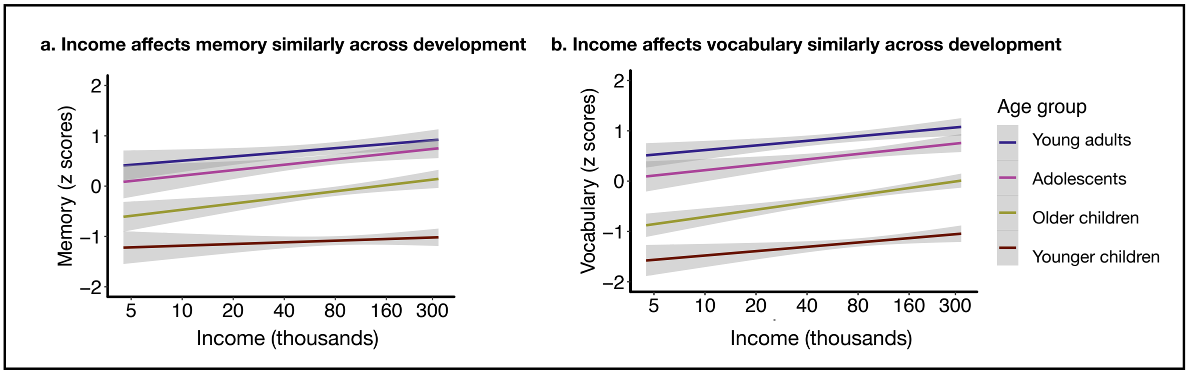

Supplementary Figure 6. The relationship between income and cognitive scores by age group. Linear regression interaction models revealed that age did not interact with income to predict a) memory ($p = 0.42$) or b) vocabulary scores ($p=.34$). Pictured above is the relationship between a) memory scores and income and b) vocabulary scores and income. In both figures, these relationships are plotted separately for young adults (blue), adolescents (pink), older children (yellow), and younger children (brown), n=690 participants. Grey shading reflects 95% confidence intervals around the mean. False discovery rate adjusted p-values for each interaction analysis are reported in the main text.

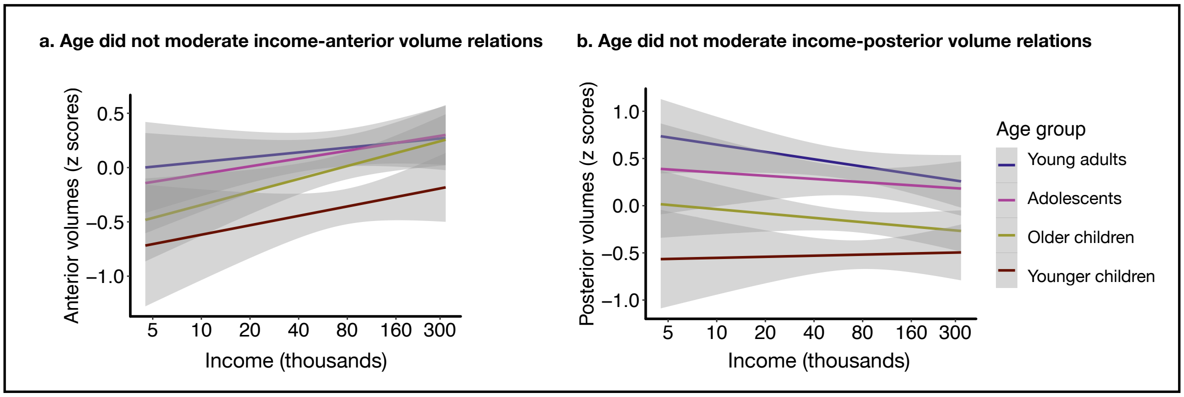

Supplementary Figure 7. The relationship between income and hippocampal subregion volumes by age group. Linear regression interaction models revealed that age did not interact with income to predict either a) anterior hippocampal volumes ($p=0.52$) or b) posterior hippocampal volumes ($p=0.50$). Displayed is the relationship between a) income and anterior hippocampal volumes and b) income and posterior hippocampal volumes. In both figures, these relationships are plotted separately for young adults (blue), adolescents (pink), older children (yellow), and younger children (brown), n=703 participants. Grey shading reflects 95% confidence intervals around the mean. False discovery rate adjusted p-values for each interaction analysis are reported in the main text.

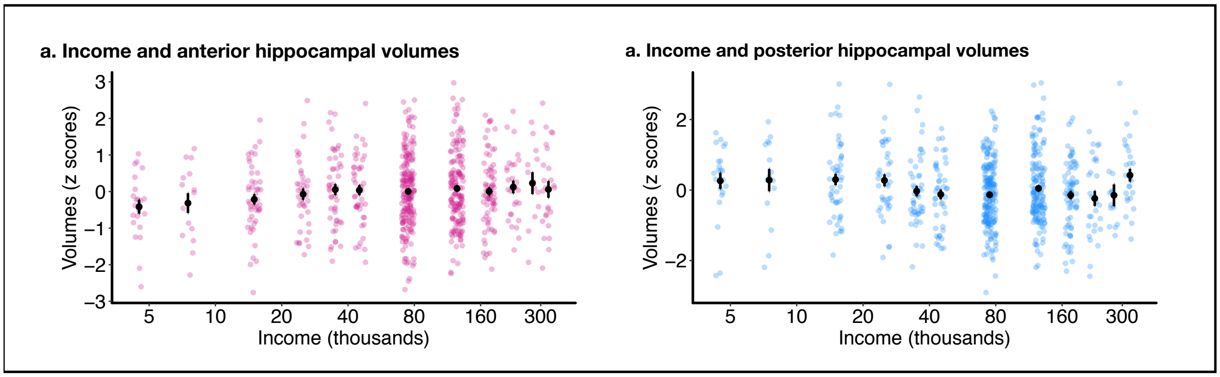

Supplementary Figure 8. The relationship between income and hippocampal subregion volumes. Linear regression interaction models (n=703 participants) revealed that income was positively related to a) anterior hippocampal volumes (p < 0.001), but b) not posterior hippocampal volumes (p = *0.15*) after controlling for age, sex, and scanner. Error bars reflect the standard error around the mean. False discovery rate adjusted p-values are presented in the text.

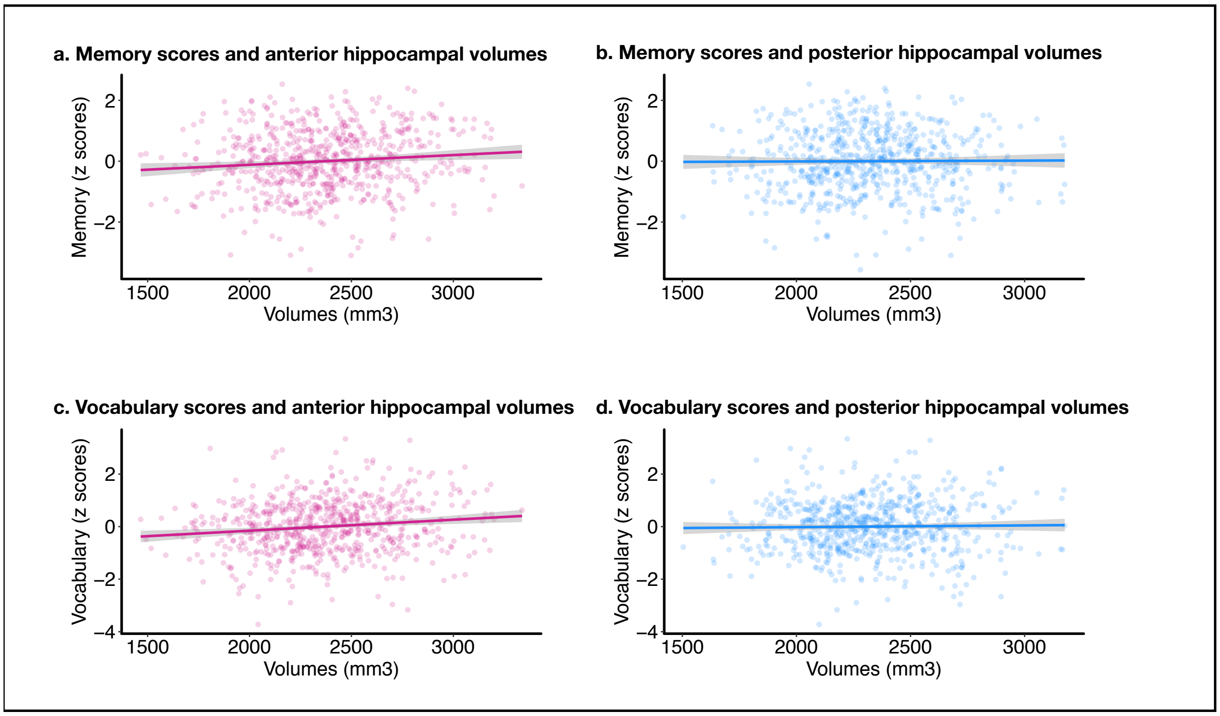

Supplementary Figure 9. The relationships between hippocampal subregion volumes and cognitive scores. Linear regressions modelling (n=690 participants) revealed that a) memory scores were positively related to anterior hippocampal volumes (p = 0.005) b) but not posterior hippocampal volumes (p = 0.83). c) Similarly, vocabulary scores were significantly related to anterior hippocampal volumes (p <0.001), but not d) posterior hippocampal volumes (p = 0.61). Z scores of cognitive scores are plotted after removing

the effects of age and sex. Grey shading reflects 95% confidence intervals around the mean. False discovery rate adjusted p-values are presented in the main text for the relationships depicted.

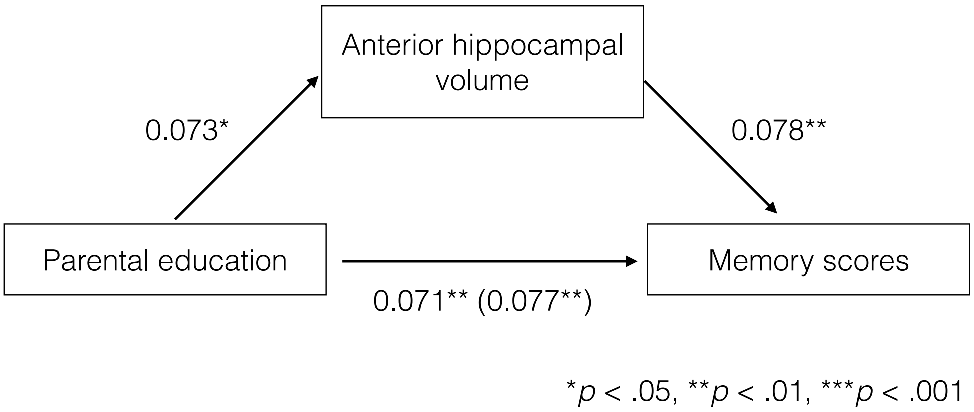

Supplementary Figure 10. Depiction of anterior hippocampal volume mediation analysis of parental education and memory score relationship. Standardized regression coefficients are reported. This analysis revealed that the positive association between parental education and memory scores was *marginally* mediated by anterior hippocampal volumes, *ab* = 0.006, *SE* =0.004, 95% CI [-0.0001, 0.01]. The total effect of parental education on memory scores was significant, *c* = 0.077, *SE* = 0.027, 95% CI [0.024, 0.13*]*, and the direct effect of parental education on memory scores was altered when the indirect path *ab* through whole hippocampal volumes was considered, *c'* = 0.071, *SE* = 0.027, 95% CI [0.019, 0.012], n = 679 participants. Confidence intervals that do not include zero reflect an effect. A false discovery rate adjusted p-value for the relationships presented above are displayed in Supplementary Tables 8,44 & 46.

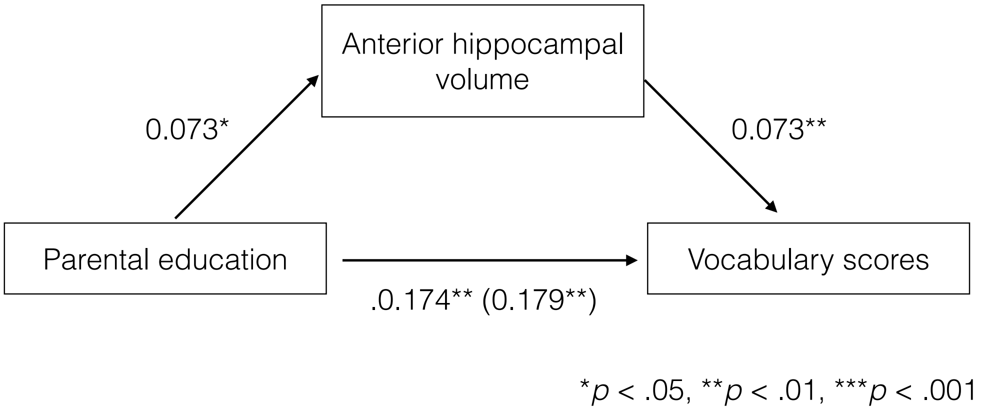

Supplementary Figure 11. Depiction of anterior hippocampal volume mediation analysis of parental education and vocabulary score relationship. Standardized regression coefficients are reported. This analysis revealed that the positive association between parental education and vocabulary scores was partially mediated by anterior hippocampal volumes, *ab* = 0.005, *SE* =0.003, 95% CI [0.001, 0.01]. The total effect of parental education on vocabulary scores was significant, *c* = 0.179, *SE* = 0.023, 95% CI [0.134, 0.22*]*, and the direct effect of parental education on vocabulary scores were altered when the indirect path *ab* through whole hippocampal volumes was considered, *c'* = 0.174, *SE* = 0.022, 95% CI [0.13, 0.22], n=679 participants. Confidence intervals that do not include zero reflect an effect. A false discovery rate adjusted p-value for the relationships presented above are displayed in Supplementary Tables 9 & 45-46.

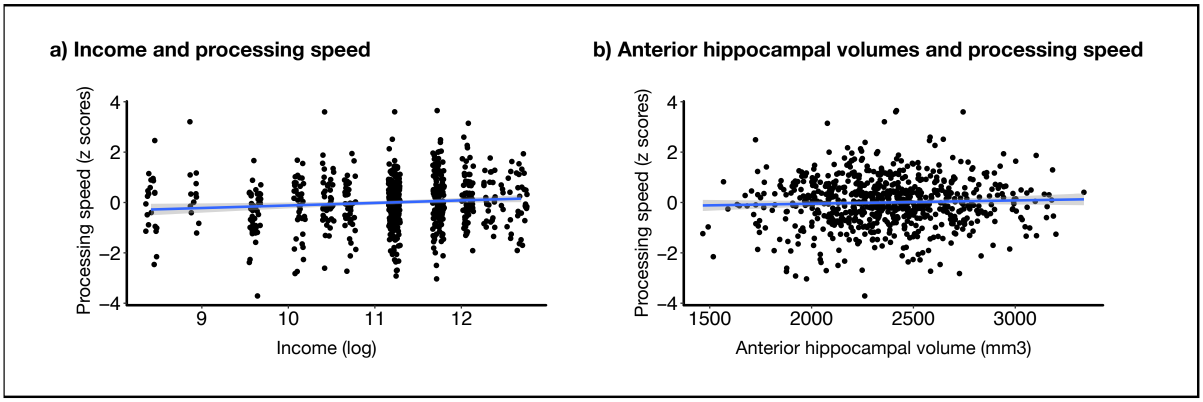

Supplementary Figure 12. The relationship between processing speed, income and anterior hippocampal volumes. Linear regressions (n=690 participants) revealed that a) family income was significantly correlated with processing speed (p = 0.008) after controlling for age and sex. b) Anterior hippocampal volumes did not correlate with processing speed after controlling for age and sex (p = 0.13). In both figures, residualized values for processing speed scores are plotted after removing variance associated with age and sex to better visualize the relationships tested. Grey shading

reflects 95% confidence intervals around the mean. A false discovery rate adjusted p-value is presented in the text.

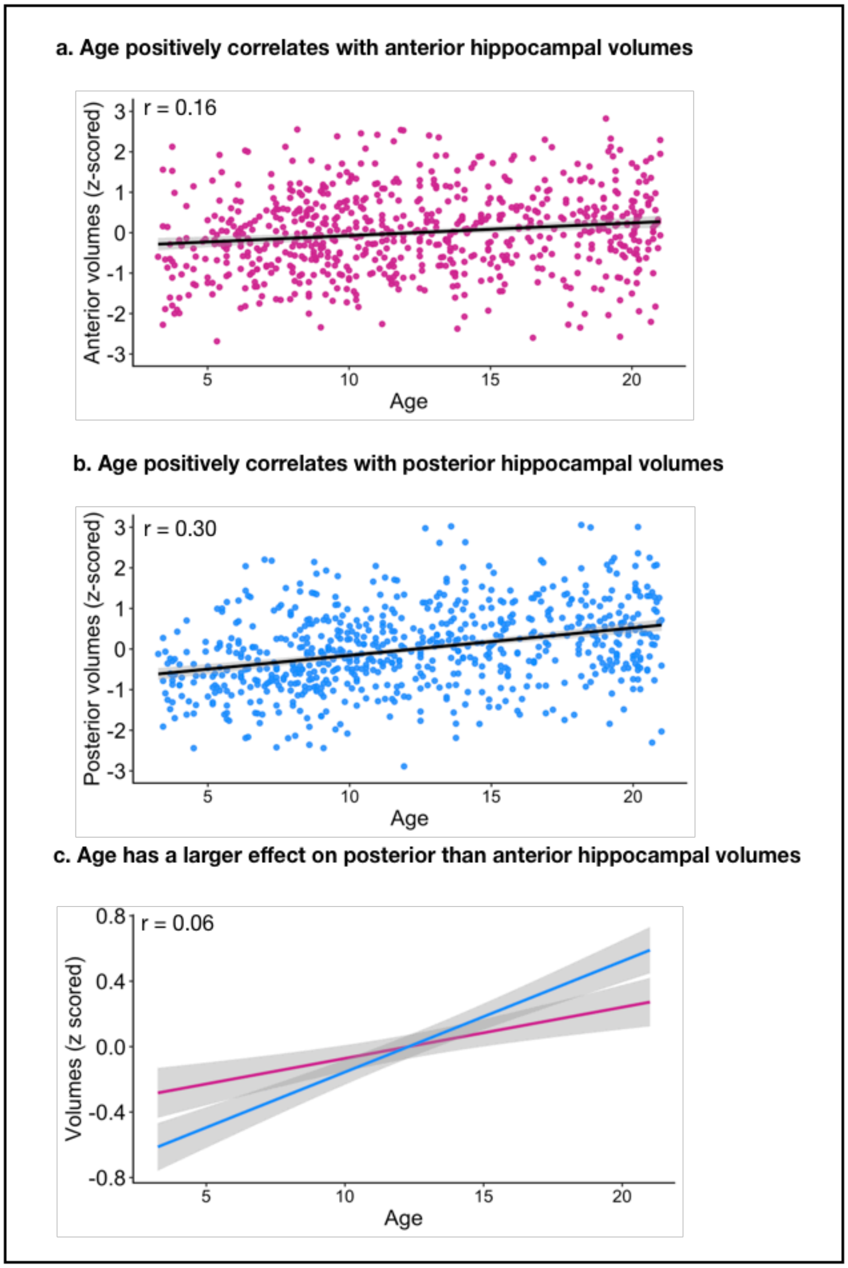

Supplementary Figure 13. Linear mixed effects modelling (n=703 participants) revealed that age had a positive relationship with a) anterior, *p < 0.001, p-adjusted: < 0.001* and b) posterior hippocampal volumes, *p < 0.001, p-adjusted < 0.001* after controlling for sex, income, and scanner. c) The relationship between age and volumes was more positive for the posterior (blue) than the anterior hippocampus (pink; interaction: p = 0.02, p-adjusted = 0.04), n=703 participants. Volumes are z-scored for ease of visualization. Grey shading reflects 95% confidence intervals around the mean. P-adjusted reflects a false discovery rate adjusted p-value.
