# Supplementary material for: Children’s family income is associated with cognitive function and volume of anterior not posterior hippocampus
Source: Nat Commun. 2020 Aug 12;11:4040. doi: 10.1038/s41467-020-17854-6 (PMC7423938; doi:10.1038/s41467-020-17854-6)
Supplement: Supplementary file 3 — Reporting Summary [file 41467_2020_17854_MOESM3_ESM.pdf]

nature research

Corresponding author(s): Alexandra Decker

Last updated by author(s): June 28, 2020

Reporting Summary

Nature Research wishes to improve the reproducibility of the work that we publish. This form provides structure for consistency and transparency in reporting. For further information on Nature Research policies, see our Editorial Policies and the Editorial Policy Checklist.

Statistics

For all statistical analyses, confirm that the following items are present in the figure legend, table legend, main text, or Methods section.

n/a	Confirmed	
☐	☒	The exact sample size (*n*) for each experimental group/condition, given as a discrete number and unit of measurement
☐	☒	A statement on whether measurements were taken from distinct samples or whether the same sample was measured repeatedly
☐	☒	The statistical test(s) used AND whether they are one- or two-sided<br>*Only common tests should be described solely by name; describe more complex techniques in the Methods section.*
☐	☒	A description of all covariates tested
☐	☒	A description of any assumptions or corrections, such as tests of normality and adjustment for multiple comparisons
☐	☒	A full description of the statistical parameters including central tendency (e.g. means) or other basic estimates (e.g. regression coefficient) AND variation (e.g. standard deviation) or associated estimates of uncertainty (e.g. confidence intervals)
☐	☒	For null hypothesis testing, the test statistic (e.g. *F*, *t*, *r*) with confidence intervals, effect sizes, degrees of freedom and *P* value noted<br>*Give P values as exact values whenever suitable.*
☒	☐	For Bayesian analysis, information on the choice of priors and Markov chain Monte Carlo settings
☐	☒	For hierarchical and complex designs, identification of the appropriate level for tests and full reporting of outcomes
☐	☒	Estimates of effect sizes (e.g. Cohen's *d*, Pearson's *r*), indicating how they were calculated

Our web collection on statistics for biologists contains articles on many of the points above.

Software and code

Policy information about availability of computer code

Data collection

Data was acquired through the Pediatric Imaging Neurocognition and Genetics study repository. There was no custom code used to collect the data.

Data analysis

Non-custom software used for analysis (with published reference)

1. Multiple Automatically Generated Templates for Different Brains algorithm was used to segment the hippocampus

Published reference: Pipitone, J., Park, M. T. M., Winterburn, J., Lett, T. A., Lerch, J. P., Pruessner, J. C., ... Alzheimer's Disease Neuroimaging Initiative. (2014). Multi-atlas segmentation of the whole hippocampus and subfields using multiple automatically generated templates. NeuroImage, 101, 494–512. https://doi.org/10.1016/j.neuroimage.2014.04.054

2. R version 3.6.3 was used to run statistical analyses

Published reference: R Core Team (2013), We used a number of packages in R. Packages and versions were the following: lme4_1.1-18-1; lmerTest_3.1-0; mediation_4.4.7; dtplyr_0.0.3; tidyverse_1.2.1; data.table_1.12.6; tidyr_1.0.0; ggplot2_3.2.0.

Non-custom software (without published reference):

3. Display image viewing and segmentation software (version 2.0) was used to visualize hippocampal labels and to manually delineate the hippocampus into anterior and posterior subregions; information on Display viewer and download instructions are accessible through the following link online: http://www.bic.mni.mcgill.ca/software/Display/Display.html

Custom code for analysing data can be found at the following link: https://github.com/alexandradecker/PING_script.

For manuscripts utilizing custom algorithms or software that are central to the research but not yet described in published literature, software must be made available to editors and reviewers. We strongly encourage code deposition in a community repository (e.g. GitHub). See the Nature Research guidelines for submitting code & software for further information.

Data

Policy information about availability of data

All manuscripts must include a data availability statement. This statement should provide the following information, where applicable:

- Accession codes, unique identifiers, or web links for publicly available datasets
- A list of figures that have associated raw data
- A description of any restrictions on data availability

The data that support the findings of this study are available from the NIMH-supported Research Domain Criteria Database. Dataset identifier(s): [10.15154/1519020]. Restrictions apply to the availability of these data, which were used under license for the current study, and so are not publicly available. Data are however available from the authors upon reasonable request and with permission of NIMH. A reporting summary for this Article is available as a Supplementary Information file.

Field-specific reporting

Please select the one below that is the best fit for your research. If you are not sure, read the appropriate sections before making your selection.

☒ Life sciences ☐ Behavioural & social sciences ☐ Ecological, evolutionary & environmental sciences

For a reference copy of the document with all sections, see nature.com/documents/nr-reporting-summary-flat.pdf

Life sciences study design

All studies must disclose on these points even when the disclosure is negative.

Sample size

We included all participants from the Pediatric Imaging and Neurocognition and Genetics study that had (1) consented to share their raw imaging data and (2) provided measures of family income, since these were the primary measures of interest in our study. In total, 703 participants were included in the final analyses. Based on prior work reporting relationships between hippocampal volumes and socioeconomic status which have smaller sample sizes than ours (Luby et al., 2009; Yu et al., (2018), we are well powered to detect significant effects.

References:
4. Luby, J. et al. The Effects of Poverty on Childhood Brain Development: The Mediating Effect of Caregiving and Stressful Life Events. JAMA Pediatr. 167, 1135–1142 (2013).
Yu, Q. et al. Socioeconomic status and hippocampal volume in children and young adults. Dev. Sci. 21, e12561 (2018).

Data exclusions

Exclusion criteria were pre-established. We excluded individuals due to: failed hippocampal segmentations (n=2), motion artifacts on MRI data (n=38), volume measures that fell 3 standard deviations from the sample mean (n=7). Failure to segment hippocampus meant there was no data for these participants and therefore these participants could not be included in the study. We excluded participants with motion artifacts or participants whose hippocampal volumes fell more than 3 SD from the sample mean to eliminate excess noise in our data.

Replication

Analysis scripts are publicly accessible online so that researchers trying to replicate our findings are aware of our analytic approach. The link the publicly accessible code is: https://github.com/alexandradecker/PING_script.

Randomization

The study was correlative and did not include an experimental manipulation. Therefore participants were not randomized to different groups. However, to control for potential confounds, we included relevant covariates in all analyses. We included age and sex as covariates in all primary and exploratory models in which cognitive scores were the dependent variable (e.g., lm(cognitive score ~ income), lm(cognitive score ~ volume)). Similarly, we included age, sex, and scanner as covariates in models in which hippocampal subregion volumes were the dependent variable. We also fit several control analyses to determine whether our effects persisted after controlling for other variables (e.g., minority status).

Blinding

For the hippocampal subfield segmentation, raters were blinded to the age, sex, and household income of participants.

Reporting for specific materials, systems and methods

We require information from authors about some types of materials, experimental systems and methods used in many studies. Here, indicate whether each material, system or method listed is relevant to your study. If you are not sure if a list item applies to your research, read the appropriate section before selecting a response.

Materials & experimental systems

n/a	Involved in the study
☒	☐ Antibodies
☒	☐ Eukaryotic cell lines
☒	☐ Palaeontology and archaeology
☒	☐ Animals and other organisms
☐	☒ Human research participants
☒	☐ Clinical data
☒	☐ Dual use research of concern

Methods

n/a	Involved in the study
☒	☐ ChIP-seq
☒	☐ Flow cytometry
☐	☒ MRI-based neuroimaging

Human research participants

Policy information about studies involving human research participants

Population characteristics

We used pre-existing data collected from the Pediatric Imaging, Neurocognition and Genetics (PING) study database (now available through the NIMH-supported Research Domain Criteria Database (RDoCdb)). Individuals between the ages of 3 and 20 were eligible to participate in the study if they had never been diagnosed with a developmental, psychiatric or neurological disorder, did not have a history of head trauma, were not born premature, and had not been exposed to drugs or alcohol prenatally for more than one trimester.

Relevant sample demographics for the present study:
Age: mean - 12.3; standard deviation - 5; range - 3-21 years
Sex: 338 female, 365 male
Annual family income: Mean- $99, 950, standard deviation- $74, 880, range- $4,500 to > $325,000.
Genetic ancestry: 10.5% African American, <1% American Indian, 8% Asian, 4.5% Hispanic, <1% Pacific Islander, 74% White

Recruitment

Participants were recruited into the PING study database using a combination of web-based, word-of-mouth and community advertising. Recruitment and data collection took place at university-based data collection sites in the United States (in and around the cities of Los Angeles, San Diego, New Haven, Sacramento, San Diego, Boston, Baltimore, Honolulu and New York). Of note, our analysis only includes participants who had the time and desire to participate in our study, and therefore there may be self-selection bias that could impact our results. While this self section bias could cause the results to be only generalizable to certain individuals, we do note that our sample is socioeconomically and ethnically diverse which could overcome some of the negative effects of the self-selection bias.

Ethics oversight

The human research protection of research subjects and institutional review board at University of California San Diego, the University of Hawaii, University of California Los Angeles, University of California Davis, Kennedy Krieger Institute at Johns Hopkins, Sackler Institute at Cornell University, the University of Massachusetts, Massachusetts General Hospital at Harvard University, and Yale University approved the study.

Note that full information on the approval of the study protocol must also be provided in the manuscript.

Magnetic resonance imaging

Experimental design

Design type

N/A - An experimental fMRI design was not used to acquire the data (neither task-based nor resting state applies).

Design specifications

N/A - An experimental fMRI design was not used to acquire the data (neither blocks nor trials apply).

Behavioral performance measures

N/A - An experimental fMRI design was not used to acquire the data (no task performed in an fMRI).

Acquisition

Imaging type(s)

Structural

Field strength

3T

Sequence & imaging parameters

The MRI protocols were standardized across participating sites. All data was acquired from a 3D T1-weighted inversion prepared RF-spoiled gradient echo scan using prospective motion correction as described previously (Brown et al., 2012; Jernigan et al., 2014).

Data was acquired in the sagittal plane with interleaved slice acquisition. All images were acquired using a 3T scanner. Scanners included Siemens, GE, or Phillips. The acquisition parameters across sites were the following:

For Siemens- TE=4.33ms, TR=2170ms, flip angle=7 degrees, voxel size = 1×1×1.2mm voxels, FoV=256; matrix size = 256x256;

For Philips- TE=3.1ms, TR=6.8ms, flip angle=8 degrees, voxel size= 1×1×1.2mm voxels, FoV=256; matrix size = 256 x 240;

For GE- TE=3.5ms, TR=8.1ms, flip angle=8 degrees, voxel size= 1×1×1.2mm voxels, FoV=256, matrix size = 256x192.

Reference:

Brown, T. T., Kuperman, J. M., Chung, Y., Erhart, M., McCabe, C., Hagler, D. J., Venkatraman, V. K., Akshoomoff, N., Amaral, D. G., Bloss, C. S., Casey, B. J., Chang, L., Ernst, T. M., Frazier, J. A., Gruen, J. R., Kaufmann, W. E., Kenet, T., Kennedy, D. N., Murray, S. S., … Dale, A. M. (2012). Neuroanatomical assessment of biological maturity. Current Biology: CB, 22(18), 1693–1698. https://doi.org/10.1016/j.cub.2012.07.002

Jernigan, T. L., Brown, T. T., Hagler, D. J., Akshoomoff, N., Bartsch, H., Newman, E., Thompson, W. K., Bloss, C. S., Murray, S. S., Schork, N., Kennedy, D. N., Kuperman, J. M., McCabe, C., Chung, Y., Libiger, O., Maddox, M., Casey, B. J., Chang, L., Ernst, T. M., … Pediatric Imaging, Neurocognition and Genetics Study. (2016). The Pediatric Imaging, Neurocognition, and Genetics (PING) Data Repository. NeuroImage, 124(Pt B), 1149–1154. https://doi.org/10.1016/j.neuroimage.2015.04.057

Area of acquisition: Whole brain

Diffusion MRI: ☐ Used ☒ Not used

Preprocessing

Preprocessing software: Prospective motion correction was used to improve image quality, reduce motion-related artifacts, and increase the reliability of quantitative measures (see White et al., 2010). Because different scanners likely have different field inhomogeneities, a gradient field nonlinearity correction was applied prior to analysis (See Jernigan et al., 2016). Multiple Automatically Generated Templates for Different Brains algorithm was used to segment the hippocampus (Pipitone et al., 2014).

Reference:
Jernigan, T. L., Brown, T. T., Hagler, D. J., Akshoomoff, N., Bartsch, H., Newman, E., Thompson, W. K., Bloss, C. S., Murray, S. S., Schork, N., Kennedy, D. N., Kuperman, J. M., McCabe, C., Chung, Y., Libiger, O., Maddox, M., Casey, B. J., Chang, L., Ernst, T. M., … Pediatric Imaging, Neurocognition and Genetics Study. (2016). The Pediatric Imaging, Neurocognition, and Genetics (PING) Data Repository. NeuroImage, 124(Pt B), 1149–1154. https://doi.org/10.1016/j.neuroimage.2015.04.057

Pipitone, J., Park, M. T. M., Winterburn, J., Lett, T. A., Lerch, J. P., Pruessner, J. C., … Alzheimer's Disease Neuroimaging Initiative. (2014). Multi-atlas segmentation of the whole hippocampus and subfields using multiple automatically generated templates. NeuroImage, 101, 494–512. https://doi.org/10.1016/j.neuroimage.2014.04.054

White, N., Roddey, C., Shankaranarayanan, A., Han, E., Rettmann, D., Santos, J., Kuperman, J., & Dale, A. (2010). PROMO: Real-time prospective motion correction in MRI using image-based tracking. Magnetic Resonance in Medicine, 63(1), 91–105. https://doi.org/10.1002/mrm.22176

Normalization: To segment the hippocampus, pre-existing manually derived hippocampal labels were warped into subject space by applying transformations estimated from nonlinear image registration (Pipitone et al., 2014; see methods).

Normalization template: Subject space. Using MAGeT-Brain segmentation, a template library is created from a subset of subject images. In particular, high resolution hippocampal labels from manually derived atlases are propagated to the subset of subject images (the template library), and then to each subject image (including subject images chosen as template images). The candidate labels for a subject are then fused into a final segmentation (See methods).

Noise and artifact removal: See above for description of prospective motion correction.

Volume censoring: N/A

Statistical modeling & inference

Model type and settings: N/A – not an functional fMRI study (neither RSA, univariate or multivariate analyses were performed).

Effect(s) tested: N/A – not a task-based fMRI study.

Specify type of analysis: ☐ Whole brain ☒ ROI-based ☐ Both

Anatomical location(s): The hippocampus was segmented using the Multiple Automatically Generated Templates for Different Brains algorithm, an automated segmentation technique, and then manually segmented into anterior and posterior subdivisions (described in the methods).

Statistic type for inference (See Eklund et al. 2016)	Linear regression or general linear mixed effects regression models were used for all analyses.
Correction	Analyses were corrected for multiple comparisons using false discovery rate correction. Adjusted p-values are presented in the Methods section of the main text.

Models & analysis

n/a	Involved in the study
☒	☐ Functional and/or effective connectivity
☒	☐ Graph analysis
☒	☐ Multivariate modeling or predictive analysis
